# Genome wide association mapping for heat tolerance in sub-tropical maize

**DOI:** 10.1186/s12864-021-07463-y

**Published:** 2021-03-04

**Authors:** Ningthaipuilu Longmei, Gurjit Kaur Gill, Pervez Haider Zaidi, Ramesh Kumar, Sudha Krishnan Nair, Vermuri Hindu, Madhumal Thayil Vinayan, Yogesh Vikal

**Affiliations:** 1 Plant Breeding and Genetics, Punjab Agricultural University, Ludhiana, Punjab India; 2 International Maize and Wheat Improvement Centre (CIMMYT), Asia Regional Office, Hyderabad, India; 3 Indian Institutes of Maize, Ludhiana, Punjab India; 4 School of Agricultural Biotechnology, Punjab Agricultural University, Ludhiana, Punjab India

**Keywords:** Association panel, Doubled haploids, Candidate genes, Genotyping by sequencing, Haplotype trend regression (HTR), Marker-trait association, SNPs

## Abstract

**Background:**

Heat tolerance is becoming increasingly important where maize is grown under spring season in India which coincide with grain filling stage of crop resulting in tassel blast, reduced pollen viability, pollination failure and barren ears that causes devastating yield losses. So, there is need to identify the genomic regions associated with heat tolerance component traits which could be further employed in maize breeding program.

**Results:**

An association mapping panel, consisting of 662 doubled haploid (DH) lines, was evaluated for yield contributing traits under normal and natural heat stress conditions. Genome wide association studies (GWAS) carried out using 187,000 SNPs and 130 SNPs significantly associated for grain yield (GY), days to 50% anthesis (AD), days to 50% silking (SD), anthesis-silking interval (ASI), plant height (PH), ear height (EH) and ear position (EPO) were identified under normal conditions. A total of 46 SNPs strongly associated with GY, ASI, EH and EPO were detected under heat stress conditions. Fifteen of the SNPs was found to have common association with more than one trait such as two SNPs viz. S10_1,905,273 and S10_1,905,274 showed colocalization with GY, PH and EH whereas S10_7,132,845 SNP associated with GY, AD and SD under normal conditions. No such colocalization of SNP markers with multiple traits was observed under heat stress conditions. Haplotypes trend regression analysis revealed 122 and 85 haplotype blocks, out of which, 20 and 6 haplotype blocks were associated with more than one trait under normal and heat stress conditions, respectively. Based on SNP association and haplotype mapping, nine and seven candidate genes were identified respectively, which belongs to different gene models having different biological functions in stress biology.

**Conclusions:**

The present study identified significant SNPs and haplotype blocks associated with yield contributing traits that help in selection of donor lines with favorable alleles for multiple traits. These results provided insights of genetics of heat stress tolerance. The genomic regions detected in the present study need further validation before being applied in the breeding pipelines.

**Supplementary Information:**

The online version contains supplementary material available at 10.1186/s12864-021-07463-y.

## Background

Maize is the third dominant cereal crop next to rice and wheat worldwide. However, in the current scenario climate change poses serious threat to maize productivity. Among the environmental stresses, heat stress (HS) is the more demanding problem which hampers maize production and there is an expectation that heat stress will further reduce the crop yields. It is predicted that at the end of twenty-first century, climate of the world will be evident to increase up to 2–4 °C. It has been forecast that upcoming catastrophe of heat stress will affect the tropical and subtropical regions of the world more based on global climate model analysis [1]. It has been described that central and eastern Asia, central North America and northern part of Indian subcontinents will be more liable regions to suffer from heat stress for growing maize and other crops [2]. It has been reported that global maize yield potential decreases to 45% by 2080s as compared to 1980s at extreme heat stress during anthesis [3].

Heat stress is a major concern to physiologists and plant breeders as nature and intensity of damage to crop varies extensively across the growing seasons due to its complex inheritance. Plant phenology, developmental phases, growth rates, yield components and final yield of plant are critically affected by thermal regimes. Other than morphological changes, several physiological and biochemical changes (photosynthetic acclimation, stalk sugar mobilization, chlorophyll content), and reproductive organ malfunctioning (low pollen viability, silk receptibility, lack of fertilization, embryo abortion, shrunken kernels) are known to be associated with HS in maize. Prolonged anthesis-silking interval, reduction in kernel set [4–8], decreased photosynthesis rate [4, 9, 10], damaged cellular membrane [11] and decreased chlorophyll content [12, 13] have been reported under heat stress. Therefore, breeding for heat tolerance in maize is crucial for sustainable productivity. Improved heat tolerance will increase resource use efficiency by reducing levels of irrigation and will increase resilience of yield in the face of the more variable and warmer climatic conditions predicted by climate change models. However, most of these traits are not used in breeding programme as selection indices because the secondary traits data recording is time consuming and requires high end instruments. Hence, alternate cost-effective methods must be worked out for robust phenotyping of these traits along with most influenced primary morphological traits like leaf firing, tassel blast and anthesis-silking interval. Exploitation of these robust phenotypic data could be done with the help of advanced genomic techniques for breeding new cultivars that are “climate resilient”.

Genomics studies along with phenotypic information can provide knowledge to the breeders that they need to make more rapid selections and application of advanced breeding strategies to produce climate-resilient crops. It is a promising tool for identifying genes or QTLs underlying heat responsive traits for translating the stress responsiveness of crop species towards marker-assisted selection approaches. Thus, analysis of genetic control of heat stress via QTL or association mapping would accelerate maize breeding programs [14]. Genome wide association studies (GWAS) would detect genomic regions controlling candidate genes by conducting statistical association between DNA polymorphisms and trait variations in diverse collection of germplasm. Genotyping-by-sequencing (GBS) platform generates millions of SNPs distributed throughout the genome in a cost-effective manner for conducting effective GWAS [15, 16]. Together with next generation sequencing and GWAS, mapping resolution of accurate position of genes/alleles/QTL has increased [17–19]. GWAS not only facilitated to identify the marker-trait association but also refined our understanding of the genetic architecture of complex quantitative traits [20, 21]. GWAS analysis have been reported in maize for flowering time [22], kernel shape, 100 seed weight [23], kernel quality [24], functional mechanisms related to drought [25] and a number of other target genes for crop improvement [26, 27], root system architecture traits [28, 29] and key traits of plant lodging and leaf angle [30]. Thus, the present study aims to (i) explore the genetic variation for heat stress responsive traits in the doubled haploid (DH) association mapping panel under normal and heat stress conditions across the environments, (ii) identify genomic regions associated with the heat tolerance traits through GWAS and (iii) identify candidate genes associated with heat stress tolerance.

## Results

### Phenotypic analysis and genetic correlation among the traits

Substantial significant variability was observed among the association mapping panel of DH lines for the agronomic traits under normal and heat stress conditions on pooled analysis of two years (Table 1). The phenotypic range was large for each trait in both the conditions and it’s indicated that there is wide range of diversity within the association mapping panel (Table 1). Frequency distribution of the lines for the investigated traits at normal and heat-stressed growth conditions are presented in an Additional file 1and 2: Figures S1 and S2. The mean performance under normal conditions was significantly different for each trait under study from the mean performance under heat stress conditions. The mean value of anthesis-silking interval (ASI), plant height (PH) and ear height (EH) was 3.6 days, 175.44 cm and 80.71 respectively, under normal conditions. While mean value of ASI, PH and EH was 5.63 days, 149.13 cm and 62.74 respectively, under heat stress conditions. Broad-sense heritability for all traits ranged from 0.41 to 0.77 under normal conditions and significantly decreased to 0.07–0.42 under heat stress (Table 1). The mean performance and statistics descriptive of days to 50% anthesis (AD) under heat stress was not presented in Table 1 as heritability was found to be zero. This may be due to opposite variation found in two years (2016 and 2017), which lead to cancellation of each other. Under heat stress, leaf firing and tassel blast were more prominent as compared to normal conditions (Data was not presented in paper). Some lines showed higher ASI and few lines had no silk emergence under heat stress conditions. Average grain yield (GY; 3.46 t/ha) was low in heat stress environment as compared to normal environment conditions (5.45 t/ha). Significant genotypic and genotypic × environment (Gen × Env) interactions were observed among the traits under study except ear position (EPO) both under non-stress and heat stress conditions. The results clearly indicated that the traits under studied were affected by high temperature.

**Table 1:** Mean and descriptive statistics of morphological traits in 662 doubled haploid association panel evaluated under normal and heat stress conditions over two growing environments

Trait	Condition	Mean	Max	Min	LSD	σ^2^ G	σ^2^ GE	H^2^
GY	Normal	5.45	6.96	3.15	2.62	0.55^***^	0.16^***^	0.51
AD		71.17	78.81	66.99	4.00	3.87^***^	0.29^***^	0.77
SD		72.83	83.31	68.51	4.45	4.57^***^	0.39^***^	0.76
ASI		1.78	3.60	0.62	3.20	0.69^***^	0.18^***^	0.51
PH		175.44	191.51	151.56	28.44	61.93^***^	6.17^***^	0.53
EH		80.71	70.93	91.84	23.19	27.87^***^	10.29^***^	0.41
EPO		0.46	0.52	0.45	0.19	0.0004	.0002	0.41
GY	Heat stress	3.46	4.58	2.55	2.57	0.35^***^	0.25^***^	0.38
SD		71.68	72.62	71.24	7.07	0.55^***^	7.45^***^	0.07
ASI		2.69	5.63	0.25	4.45	1.07^***^	0.35^***^	0.42
PH		149.13	153.13	144.07	29.04	15.14^***^	42.45^***^	0.17
EH		62.74	69.53	55.41	22.03	18.11^***^	16.89^***^	0.31
EPO		0.42	0.47	0.38	0.11	0.0004	.0002	0.32

^*^ = 0.05% significant, ^***^ = 0.001% significant, *σ*^*2*^ *G* Genotype variance, *σ*^*2*^ *GE* Genotype x Environment variance, *Max* Maximum, *Min* Minimum, *H*^*2*^ broad-sense heritability, *LSD* Least significant difference, *GY* Grain yield, *AD* Days to 50% anthesis, *SD* Days to 50% silking, *ASI* Anthesis-silking interval, *PH* Plant height, *EH* Ear height and *EPO* Ear position

The traits viz.*,* AD, SD (days to 50% silking) and ASI showed negative and significant relationship with GY in both normal and heat stress conditions (Table 2). This illustrated that prolonged ASI (> 5 days) will results in increase in grain yield reduction. Under normal and heat stress conditions, the traits: PH, EH and EPO displayed positive and significant association with GY. The correlation analysis inferred significant association among the traits except AD and SD that showed negative and non-significant association with EPO under normal conditions.

**Table 2:** Genetic correlation among traits under across normal environment (above diagonal) and heat stress conditions (below diagonal)

Trait	AD	SD	ASI	PH	EH	EPO	GY
AD	1	0.87^***^	− 0.04^***^	0.32^***^	0.38^***^	−0.05	− 0.18^***^
SD	–	1	0.22^***^	0.26^***^	0.31^***^	−0.08	− 0.27^***^
ASI	–	0.35^***^	1	−0.44^***^	−0.41^***^	− 0.53^***^	−0.76^***^
PH	–	0.99^**^	−0.12^**^	1	0.72^***^	0.31^***^	0.40^***^
EH	–	0.82^***^	−0.31^***^	0.95^***^	1	0.88^***^	0.41^***^
EPO	–	0.34^***^	−0.39^***^	0.75^***^	0.95^***^	1	0.35^***^
GY	–	−0.99^***^	−0.76^***^	0.39^***^	0.31^***^	0.22^***^	1

^*^ = 0.05% significant, ^**^ = 0.01% significant, ^***^ = 0.001% significant, − = no information, *AD* Days to 50% anthesis, *SD* Days to 50% silking, *ASI* Anthesis-silking interval, *PH* Plant height, *EH* Ear height, *EPO* Ear position and *GY* Grain yield

### Principal component analysis

The objective of principal component analysis (PCA) is to measure the variance that exists in genome-wide markers. The first four principal components (PCs) explained 28.7% of the total variance (Additional file 3: Figure S3). The PC1, PC2, PC3 and PC4 explained 16.2, 6.8, 3.6 and 2.7% of the total variance, respectively. The panel with 662 DH lines revealed only a moderate population structure with the first four principal components using genome-wide markers.

### Genome wide association mapping based on the SNPs

**Fig. 1:**
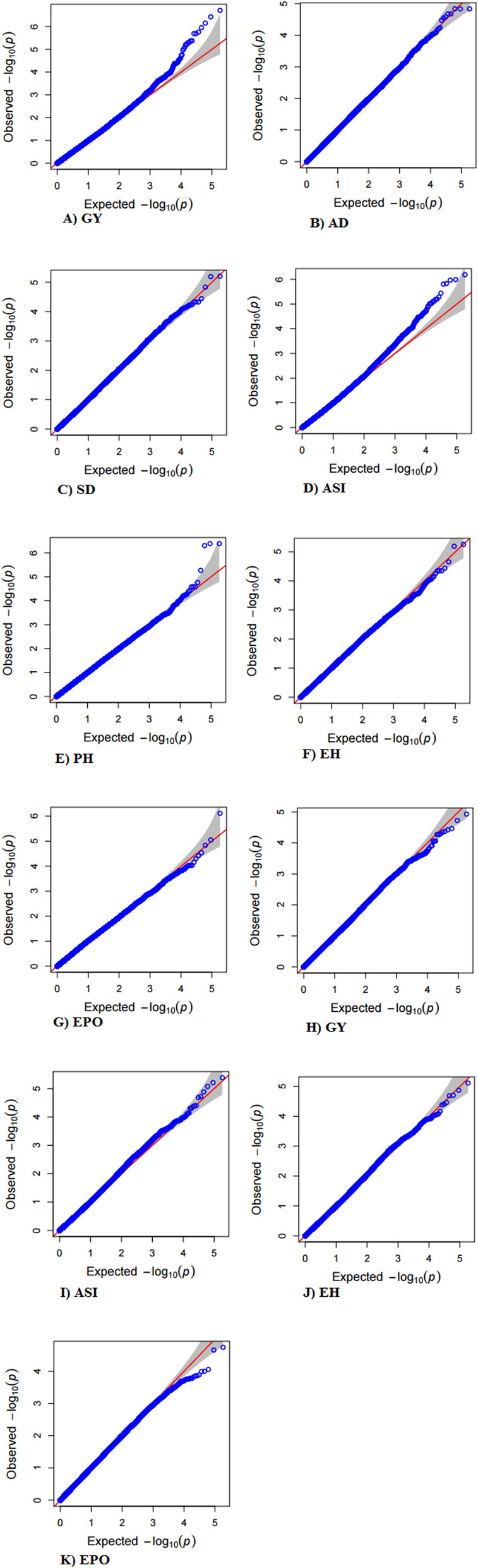
Quantile-qantile (Q-Q)-plots showing inflation of estimated –log10 *P*-values on the X-axis versus observed –log10 *P*-values on the Y-axis for the traits using MLM model under pooled normal (A-G) and heat stress conditions (H-K). *GY* Grain yield, *AD* Days to 50% anthesis, *SD* Days to 50% silking, *ASI* Anthesis-silking interval, *PH* Plant height, *EH* Ear height and *EPO* Ear position

**Fig. 2:**
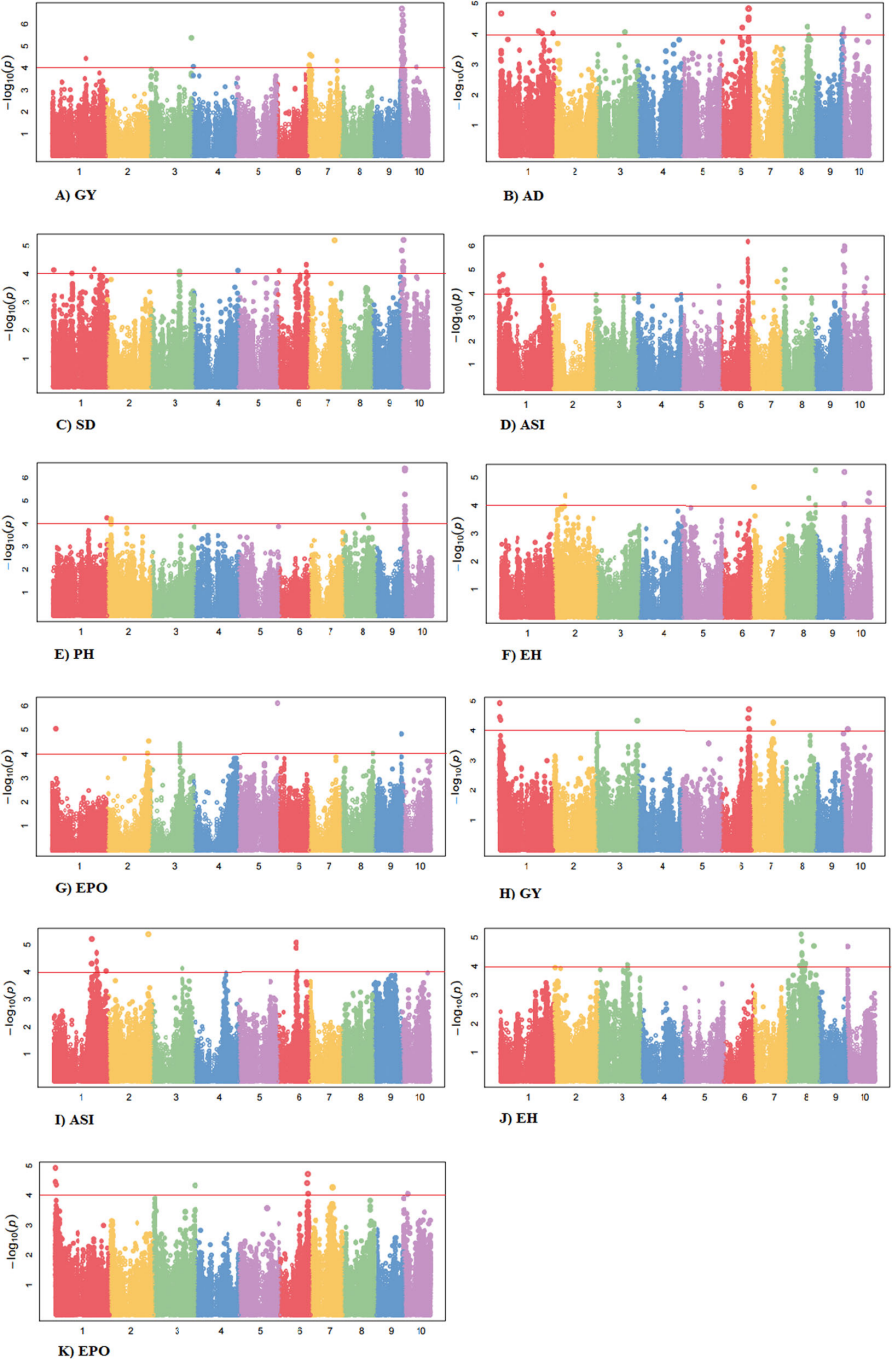
Manhattan plot from MLM for the different traits under normal (A-G) and heat stress (H-K) conditions plotted with the individual SNPs of all chromosomes on the X-axis and –log10 P-values of each SNP in the Y-axis. The different color represents the 10 chromosomes of maize. *GY* Grain yield, *AD* Days to 50% anthesis, *SD* Days to 50% silking, *ASI* Anthesis-silking interval, *PH* Plant height, *EH* Ear height and *EPO* Ear position

The traits which showed heritability greater or equal to 0.20 were used for genome-wide association studies. A total of 187,000 SNPs was employed for GWAS analysis. The quantile-quantile (QQ)-plot was used to assess the significance of SNPs at a threshold level using MLM model. The QQ-plot generated for all the traits across environments is represented in Fig. 1a-g (under normal conditions) and Fig. 1h-k (under heat stress conditions). From the QQ-plot, it was evident that most of the SNPs were not associated with the traits under study. The presence of spurious associations was shown by deviation from the diagonal line due to population structure and familial relatedness while the SNPs on the upper section of the graph deviated from the diagonal were most likely to be associated with the traits. A total of 130 highly significant marker-trait associations (P = ≤10^− 5^) were observed for the target traits under normal conditions (Table 3, Fig. 2a-g, Additional file 4: Table S1). Out of which, twenty-five major SNPs were associated at *p*-value 10^− 5^ to 10^− 7^ for GY localized on chromosomes 1, 3, 7 and 10 (Fig. 2a). The phenotypic variation expressed by these SNPs ranged from 21.90 to 23.23%. Seventeen significant SNPs detected on chromosomes 1, 3, 6 and 10 were related to AD at *p*-value 10^− 5^ and phenotypic variation explained by these SNPs ranged from 45.45 to 45.78% (Fig. 2b). Nineteen SNPs for SD trait identified on chromosomes 1, 3, 4, 6, 7 and 10 accounting phenotypic variation from 36.69 to 39.21% (Fig. 2c) whereas twenty-five significant SNPs present on chromosomes 1, 6, 8, and 10 were identified for ASI at p-value 10^− 5^ to10^− 7^ (Fig. 2d). The proportion of variation explained by these SNPs ranged from 20.94 to 21.89%. Nineteen and fourteen significant SNPs were observed for PH and EH present on chromosomes 1, 2, 8 & 10 and on chromosomes 2, 7, 8 & 10, respectively, attributing phenotypic variation from 23.10 to 27.03% (Fig. 2e, f). EPO showed association with eleven significant SNPs localized on chromosomes 1, 2, 3, 5, 8 and 9 with variance of 4.39 to 5.85% (Fig. 2g).

**Table 3:** Summary of SNPs associated with different traits as detected by genome wide association studies (GWAS) under normal and heat stress conditions using 662 doubled haploid association panel

Condition	Trait	N^o^ s	Chromosome (N^o^ s)	P-value range	PV (%)
Normal	GY	25	1 (1), 3 (1), 7 (2), 10 (21),	10^−5^ – 10^−7^	21.9–23.23
	AD	17	1 (6), 3 (1), 6 (6), 8 (1), 10 (3)	10^− 5^	45.45–45.78
	SD	19	1 (3), 3 (1), 4 (1), 6 (5),7 (1), 10 (8)	10^−5^ – 10^− 6^	36.69–39.21
	ASI	25	1 (4), 6 (11), 8 (2), 10 (8)	10^−5^ – 10^−7^	20.94–21.89
	PH	19	1 (1), 2 (3), 8 (2), 10 (13)	10^−5^ – 10^−6^	25.75–27.03
	EH	14	2 (3), 7 (1), 8 (3), 10 (7)	10^−5^ – 10^− 6^	23.10–23.79
	EPO	11	1 (1), 2 (3), 3 (4), 5 (1), 8 (1), 9 (1)	10^−5^ – 10^−7^	4.39–5.85
Heat stress	GY	12	1 (3), 3 (1), 6 (3), 7 (3), 10 (2)	10^−5^	18.14–18.65
	ASI	17	1 (12), 2 (1), 3 (1), 6 (3)	10^−5^ – 10^−6^	27.81–28.53
	EH	14	3 (1), 8 (12), 10 (1)	10^−5^ – 10^−6^	25.67–26.27
	EPO	3	8 (3)	10^−5^	33.44–35.69

*N*^*o*^ *s* Number of significant SNP-based associations, The value in parenthesis indicates the number of SNPs detected in that particular chromosome, *PV (%)* Percentage of the phenotypic variation range explained by SNP markers, *GY* Grain yield, *AD* Days to 50% anthesis, *SD* Days to 50% silking, *ASI* Anthesis-silking interval, *PH* Plant height, *EH* Ear height and *EPO* Ear position

Under heat stress, a total of 46 SNPs significantly associated with the yield contributing traits were identified (Table 3, Fig. 2h-k, Additional file 4: Table S1). Twelve SNPs present on chromosomes 1, 3, 6, 7 and 10 for GY were detected (Fig. 2h). The phenotypic variation documented by these SNPs ranged from 18.14 to 18.65%. In case of ASI at *p*-value 10^− 5^ to10^− 6^ seventeen significant SNPs were identified on chromosomes 1, 2, 3 and 6 and the proportion of variation ranged from 27.81 to 28.53% (Fig. 2i). On chromosomes 3, 8 and 10 fourteen significant SNPs were found for EH explaining phenotypic variation from 25.67 to 26.27% (Fig. 2j). Only three significant SNPs on chromosome 8 were detected for EPO at p-value 10^− 5^ with proportion of variation from 21.56 to 21.95% (Fig. 2k). These results showed so many haplotypes in SNPs marker-trait association studies because of DH population as only one recombination occurred during generation of DHs.

From breeding point of view, SNPs that were associated with more than one trait gains more importance. In present study, 15 SNPs were co-localized for multiple agronomic traits and 9 SNPs were present within the gene model (Table 4). Out of which, six SNPs (S6_156,527,428, S6_156,527,431, S6_156,527,432, S1_4,752,039, S6_156,527,380 and S1_228,565,627) were found commonly associated with AD and SD as evident from strong correlation between these two traits (Table 2). The proportion of phenotypic variation explained by these SNPs ranged from 38.71 to 45.78%, respectively. The SNP, S10_7,132,845 was linked with AD, SD and GY. The phenotypic variation accounted by this SNP ranged from 22.91–45.51%. Two SNPs (S10_8,852,411 and S10_9,473,175) showed association with ASI and SD; one SNP (S10_1,888,234) with EH and GY; two SNPs (S10_1,905,273 and S10_1,905,274) with PH, EH and GY; one SNP (S10_10,826,645) with GY and SD; and two SNPs (S10_1,148,841 and S10_1,883,817) with GY and PH. Among these associations for agronomic traits, favorable associations have been often observed on chromosomes 6 and 10 suggesting the presence of either a common gene or gene clusters responsible for heat stress tolerance.

**Table 4:** List of significant SNPs associated with more than one trait under normal conditions

SNP	Chr	Position (bp)	Trait	P-value	MAF	PV (%)	Gene model
S6_156,527,428	6	156,527,428	AD +SD	10^−5^	0.21	38.82–45.78	
S6_156,527,431	6	156,527,431	AD + SD	10^−5^	0.21	38.82–45.78	
S6_156,527,432	6	156,527,432	AD + SD	10^−5^	0.21	38.82–45.78	
S1_4,752,039	1	4,752,039	AD +SD	10^−5^	0.16	38.74–45.71	
S6_156,527,380	6	156,527,380	AD + SD	10^−5^	0.19	38.71–45.63	
S1_228,565,627	1	228,565,627	AD + SD	10^−5^	0.27	38.76–45.45	GRMZM5G877815
S10_7,132,845	10	7,132,845	AD + SD + GY	10^−5^ – 10^−7^	0.10	22.91–45.51	GRMZM2G031624
S10_8,852,411	10	8,852,411	ASI + SD	10^−5^ – 10^−6^	0.22	21.77–38.78	GRMZM2G438176
S10_9,473,175	10	9,473,175	ASI + SD	10^−5^ – 10^−6^	0.23	21.76–38.72	GRMZM2G048850
S10_1,888,234	10	1,888,234	EH + GY	10^−5^ – 10^−6^	0.45	22.22–23.75	AC198366.3_FGT004
S10_1,905,273	10	1,905,273	EH + GY + PH	10^−5^ – 10^−7^	0.46	22.36–27.04	GRMZM2G104620
S10_1,905,274	10	1,905,274	EH + GY + PH	10^−5^ – 10^−7^	0.46	22.36–27.04	GRMZM2G104620
S10_10,826,645	10	10,826,645	GY + SD	10^−6^	0.11	22.79–39.21	GRMZM2G418432
S10_1,148,841	10	1,148,841	GY + PH	10^−5^ – 10^−6^	0.35	22.68–25.87	GRMZM2G057557
S10_1,883,817	10	1,883,817	GY + PH	10^−5^ – 10^−6^	0.49	21.91–26.43	GRMZM2G317287

*Chr* Chromosome, *Position (bp)* Position of SNP in base pairs, P-value = Significance threshold level, *MAF* Minor allele frequency, *PV (%)* Percentage of the phenotypic variation range explained by SNP markers, *GY* Grain yield, *AD* Days to 50% anthesis, *SD* Days to 50% silking, *ASI* Anthesis-silking interval, *PH* Plant height, *EH* Ear height and *EPO* Ear position

### Genome wide association based on haplotypes

Haplotype trend regression (HTR) was further performed for the GWAS significant SNPs (P = < 10^− 3^). A total of 955 SNPs associated with ASI (301), EPO (176), EH (267) and GY (211) under heat stress were included for haplotype block analysis. Likewise, 1596 SNPs related to PH (176), AD (186), SD (245), ASI (377), EPO (157), EH (170) and GY (285) under normal conditions were implemented for haplotype block analysis. A total of 125 (each containing SNPs from 2 to 13) and 85 haplotype blocks (each containing SNPs from 2 to 22) were identified, respectively, under normal and heat stress conditions (Table 5). A total of 33, 14, 21, 17, 11, 18 and 11 haplotypes were associated with GY, AD, SD, ASI, PH, EH and EPO respectively, under normal conditions. Similarly, 21 haplotypes each for GY and ASI were detected whereas 29 and 14 haplotypes were observed for EH and EPO respectively, under heat stress conditions. The maximum phenotypic variance was explained by PH (21.10%) followed by AD (15.64%) and GY (15.00%) under normal conditions while ASI (17.63%) showed highest phenotypic variation followed by EH (11.63%) under heat stress.

**Table 5:** Summary of haplotypes associated with the traits under normal and heat stress conditions

Condition	Trait	N^o^ h	Chromosome (N^o^ h)	PV (%)
Normal	GY	33	3 (3), 4 (1), 6 (1), 7 (8), 9 (1), 10 (21)	2.31–15.00
	AD	14	1 (3), 2 (1), 3 (1), 5 (1), 6 (1), 8 (4), 9 (1), 10 (1)	1.90–15.64
	SD	21	1 (5), 3 (2), 4 (1), 5 (3), 6 (3),7 (1), 8 (3), 10 (3)	1.87–9.46
	ASI	17	1 (5), 3 (2), 4 (3), 6 (2), 8 (1), 9 (1), 10 (3)	2.81–11.87
	PH	11	1 (1), 2 (1), 4 (1), 5 (1), 10 (7)	1.82–21.10
	EH	18	2 (3), 3 (1), 4 (1), 5 (2), 7 (1), 8 (7), 10 (3)	2.29–13.59
	EPO	11	1 (1), 2 (3), 3 (4), 5 (1), 8 (1), 9 (1)	1.85–4.48
Heat stress	GY	21	1 (8), 3 (4), 7 (6)	2.37–10.28
	ASI	21	1 (13), 4 (2), 6 (3), 7 (2), 9 (1)	1.95–17.63
	EH	29	3 (6), 8 (21), 9 (1), 10 (1)	2.02–11.63
	EPO	14	3 (2), 5 (1), 7 (1), 8 (10)	2.03–9.97

*N*^*o*^ *h* Number of significant haplotypes based associations, The value in parenthesis indicates the number of haplotypes detected in that particular chromosome, *PV (%)* Percentage of the phenotypic variation range explained by haplotypes markers, *GY* Grain yield, *AD* Days to 50% anthesis, *SD* Days to 50% silking, *ASI* anthesis-silking interval, *PH* Plant height, *EH* Ear height and *EPO* Ear position

A total of 20 and 6 significant haplotypes were detected, which control more than one trait under normal and heat stress conditions, respectively (Tables 6). Three haplotypes viz. Hap_4, Hap_4.1 and Hap_4; and Hap_10.2, Hap_10.3, Hap_10.4 were commonly associated with SD, ASI and GY; and PH, EH and GY, respectively, under normal conditions. Haplotypes, Hap_10.1 and Hap_10.2; Hap_10.1 and Hap_10.3; and Hap_8 and Hap_8.7 were documented for PH and GY; EH and GY; and EPO and EH, respectively, whereas eight haplotypes (Hap_1.1, Hap_1.3, Hap_5.2, Hap_5.3, Hap_6, Hap_6.3, Hap_8.3 and Hap_8.2) were related to AD and SD under normal conditions. Under heat stress, six blocks (Hap_3.1, Hap_3.2, Hap_8.14, Hap_8.2, Hap_8.16 and Hap_8.3) present on chromosomes 3 and 8 were commonly associated with EH and EPO. Among these associations for agronomic traits, favorable associations were often observed on chromosomes 8 and 10 which may be due to pleiotropic effect or presence of gene clusters that are responsible for heat stress tolerance.

**Table 6:** List of significant haplotypes associated more than one trait under normal and heat stress conditions

Condition	Haplotype blocks	Chr.	Trait	Marker used	Alleles/Haplotypes	R squared	Bonferroni P	Gene model
Normal	Hap_4	4	GY	S4_4748055, S4_4748074	2	0.065935	1.73E-08	GRMZM2G583593
	Hap_4.1		ASI	S4_4748055, S4_4748074	2	0.053516	9.75E-07	
	Hap_4		SD	S4_4748055, S4_4748074	2	0.019148	0.029166	
	Hap_10.2	10	GY	S10_1,148,841, S10_1280099	2	0.089652	8.79E-09	GRMZM2G057557
	Hap_10.1		PH	S10_1,148,841, S10_1280099	2	0.093807	1.53E-09	
	Hap_10.3	10	GY	S10_1902587, S10_1902592, S10_1902657	2	0.134391	5.94E-17	
	Hap_10.1		EH	S10_1902587, S10_1902592, S10_1902657	2	0.086085	1.07E-10	
	Hap_10.4	10	GY	S10_1905237, S10_1905245, S10_1,905,273, S10_1,905,274	3	0.071581	3.63E-09	GRMZM2G104620
	Hap_10.3		PH	S10_1905237, S10_1905245, S10_1,905,273, S10_1,905,274	3	0.103564	6.45E-14	
	Hap_10.2		EH	S10_1905237, S10_1,905,273, S10_1,905,274	3	0.06899	5.25E-09	
	Hap_1.1	1	AD	S1_225496598, S1_225575695	3	0.144835	1.58E-20	GRMZM2G056594
	Hap_1.3		SD	S1_225496598, S1_225575695	3	0.086711	8.38E-12	
	Hap_5.2	5	AD	S5_213280266, S5_213280287	2	0.017354	0.01968	GRMZM2G018484
	Hap_5.3		SD	S5_213280266, S5_213280287	2	0.023856	0.00298	
	Hap_6	6	AD	S6_156,527,380, S6_156527416, S6_156,527,428, S6_156,527,431, S6_156,527,432, S6_156527456	2	0.038081	6.56E-05	
	Hap_6.3		SD	S6_156,527,380, S6_156527416, S6_156,527,428, S6_156,527,431, S6_156,527,432, S6_156527456	2	0.035333	0.000222	
	Hap_8.3	8	AD	S8_146060437, S8_146060465, S8_146060468, S8_146060469, S8_146060472, S8_146060474, S8_146060484, S8_146060492	3	0.156355	8.25E-23	GRMZM2G109144
	Hap_8.2		SD	S8_146060437, S8_146060465, S8_146060468, S8_146060469, S8_146060472, S8_146060474, S8_146060484, S8_146060492	3	0.085053	7.68E-12	
	Hap_8	8	EPO	S8_170952700, S8_170952712	3	0.027939	0.000604	GRMZM2G379128
	Hap_8.7		EH	S8_170952700, S8_170952712	3	0.135859	3.60E-20	
Heat stress	Hap_3.1	3	EH	S3_135466614, S3_135501902, S3_135505933, S3_135695557, S3_135695608	2	0.051163	4.21E-06	
	Hap_3.2		EPO	S3_135466614, S3_135501902, S3_135695557	3	0.04404	2.05E-05	
	Hap_8.14	8	EH	S8_92967095, S8_92967143, S8_92967144, S8_92967146, S8_92967148, S8_92967149, S8_92967304	2	0.039025	0.000159	
	Hap_8.2		EPO	S8_92967095, S8_92967143, S8_92967144, S8_92967146, S8_92967148, S8_92967149	2	0.027798	0.002761	
	Hap_8.16	8	EH	S8_102137856, S8_102137869	2	0.081829	3.94E-11	GRMZM2G887068
	Hap_8.3		EPO	S8_102137856, S8_102137869	2	0.082216	2.89E-11	

*Chr* Chromosome, *GY* Grain yield, *AD* Days to 50% anthesis, *SD* Days to 50% silking, *ASI* anthesis-silking interval, *PH* Plant height, *EH* Ear height and *EPO* Ear position

### Candidate genes associated with the target traits

Based on SNP and haplotypes genome wide association mapping, 9 and 7 candidate genes were identified and annotated with different functions (Additional file 5:Table S2). Out of which, GRMZM5G877815 detected for AD and SD, GRMZM2G031624 for AD, SD and GY, GRMZM2G438176 and GRMZM2G048850 for ASI and SD, AC198366.3_FGT004 for EH and GY, GRMZM2G104620 for EH, GY and PH, GRMZM2G418432 for GY and SD, GRMZM2G057557 and GRMZM2G317287 for GY and PH. The SNPs (S4_4748055, S10_1,148,841, S10_1902587, S1_225496598, S5_213280266, S8_146060437, S8_170952700 and S8_102137856) involved in haplotype blocks were co-localised within the gene model: GRMZM2G583593, GRMZM2G057557, GRMZM2G104620, GRMZM2G056594, GRMZM2G018484, GRMZM2G109144, GRMZM2G379128 and GRMZM2G887068. Out of 7 candidate genes, GRMZM2G583593 was detected for GY, ASI and SD; GRMZM2G056594, GRMZM2G018484 and GRMZM2G109144 for AD and SD while GRMZM2G379128 and GRMZM2G887068 for EP and EPO.

## Discussion

Heat stress due to rise in temperature beyond optimum is a major threat for sustainable production of crops and shortening of cropping periods [31]. Climatic model analysis delineates central and eastern Asia, central North America and northern part of Indian subcontinent as the major heat stress prone regions. Maize is highly sensitive to heat stress during the months of April end and May which coincide with grain filling of the crop, and results in tassel blast, reduced pollen viability, fertilization failure and barren ears that causes devastating yield losses [32]. Since, heat tolerance is a polygenic trait and is more prone to genotype-environment interactions, agronomic interventions could do a very little to alleviate this stress. Breeding for heat stress tolerance is the most economical approach to challenge climate change globally [33, 34]. To meet this challenge advances in genomics assisted pre-breeding approaches by identifying superior alleles from well adapted genetic resources and their utilization in breeding programs is now widely recommended [35]. We conducted GWAS to map QTLs and identified SNP markers associated with heat tolerance using DH mapping panel for various agronomic traits under normal and heat stress conditions. Exploiting the variability and identification of significant marker-trait associations based on GWAS might provide a platform for heat stress tolerance in maize breeding through marker-assisted introgression of heat tolerance alleles into elite germplasm.

All the traits were adversely affected by high temperature as indicated by significant reduction in PH, EH and GY whereas ASI increased dramatically under heat stress conditions as compared to normal conditions in the present investigation. This might be due to decreased partitioning of assimilate to the ear and low pollen viability. The mechanisms of increased ASI under heat stress conditions have been discussed by Cicchino et al. [36]. Reduction of GY up to 70% under heat stress has been reported by Khodarahmpour and Choukan [37] which might be due to low pollen viability, silk receptivity and longer ASI duration. Similar observations have been made in our study. Leaf firing and tassel blast were observed among the panel under heat stress as heat waves resulted in cell injury which led to chlorosis and death of the tissue that affected the photosynthetic apparatus and reduction in GY [38]. Broad-sense heritability was low for the traits under heat stress as compared to moderate under normal conditions. So, it could be hypothesized that genotypic variability and genotype-environment interactions played an important role for the expression of a particular trait under heat stress and normal conditions. Previous results depicted significant high heritability for traits viz.*,* GY, AD, SD, ASI, leaf firing and tassel blast for hybrids [39] and for inbred lines [40] under heat stress conditions. However, our results strongly agreed with the findings of Noor et al. [40]. We found low to high positive (r between 0.22–0.99) and significant correlation between PH and SD (0.99), SD and EH (0.82), EH and PH (0.95), and PH and EPO (0.95) under heat stress whereas EH and PH (0.72), EH and EPO (0.88), and AD and SD (0.87) showed strong positive association under normal conditions. The positive significant associations were observed among traits suggested that these traits might share some common genomic regions. Similar significant relationships for agronomic traits under normal and stress conditions were reported in previous studies [26, 28, 41]. Moderate to strong negative correlations was observed between ASI and other traits (PH, EH, EPO, GY) under both normal and heat stress conditions.

The associating mapping panel showed moderate principal components within it. Warburton et al. [42] also observed large amount of diversity within, rather than between source populations due to the heterogeneous nature of CIMMYT populations from which most of the sub-tropical and tropical lines have been derived. The moderate structure that was observed in the present study panel may be due to the inclusion of DH lines derived from crosses of selected inbreds. Before proceeding for GWAS analysis, it is necessary to use appropriate models to control false associations which could confound association mapping due to the population structure and the kinship relationship within the panel [43].

In present study, a set of 176 SNPs were associated with the various agronomic traits under normal and heat stress conditions. Out of these, some SNPs existed within different gene models whose genetic role is associated with heat stress mechanism. It has been postulated that genes associated with the target traits significantly could be resequenced from contrasting lines to identify favorable alleles for trait improvement and SNP/InDELs could be potentially converted to simple PCR-based markers to follow MAS in molecular breeding [44, 45]. Several studies have been made in maize for identification of associated SNPs with a particular trait using GWAS analysis [25, 26, 30, 41]. We identified 130 SNPs associated with different traits distributed over all the 10 maize chromosomes but most of them localized on chromosomes 3, 8 and 10 under normal conditions. The overall range of phenotypic variance explained by each SNP was from 4.39–45.78%. A total of 15 SNPs were colocalized with multiple traits and nine of them were present within the candidate genes. The largest SNPs hotspot regions were observed on chromosome 10 significantly associated with AD, SD, PH, EH, ASI and GY traits followed by chromosome 8 associated with AD, ASI, EH and PH attributes under normal conditions. Most significant SNP markers associated with ASI and GY were localized on chromosomes 1 and 6 under heat stress. The results clearly revealed that different genomic regions are involved under the normal and heat stress conditions for the expression of a particular trait. It could be concluded that there is switch off mechanism for heat stress tolerance under stress conditions as compared to normal conditions. Xue et al. [26] identified 42 SNPs located in 33 genes associated with 126 traits × environment × treatment combinations and three SNPs were co-localized to drought-related QTL regions. One of the genes GRMZM2G125777 that encodes NAC domain containing protein 2, a transcription factor expressed in different tissues, was strongly associated with ear relative position, hundred kernel weights and timing of male and female flowering [26]. Similarly, Wang et al. [41] identified 206 significant SNPs associated with 115 candidate genes for drought tolerance and related traits. Zaidi et al. [28] revealed 67 significantly associated SNPs for root structural traits whereas Frey et al. [46] revealed 607 heat responsive genes as well as 39 heat tolerance genes. Compared to these studies, our results depicted only nine candidate genes because we had looked only for the colocalized SNPs. It might be possible to predict a greater number of candidate genes if we considered all the 130 SNPs. On the other hand, under heat stress we identified 46 SNPs associated with ASI, EH, EPO and GY distributed on chromosomes 1, 2, 3, 6, 7, 8 and 10 with variance of 18.14–35.69%. It is surprised to conclude that none of the SNP colocalized with multiple traits under heat stress.

Haplotype-based analysis for identifying marker-phenotype associations is beneficial for the genetic dissection of loci underlying complex traits [47, 48]. As compared to individual SNP-based associations, haplotype-based analysis could lessen the number of multiple evaluations since haplotype could cluster SNPs from the LD configuration [30]. Also, in this study, as DH lines formed from F_1_ source populations were used in the AM panel, and hence the LD blocks are expected to be large in the panel, which can have an impact on mapping resolution. Haplotype analysis, which takes into consideration this LD structure, will be extremely useful in such cases. In our study, a total of 210 haplotypes were identified at Bonferroni P = ≤0.05 for the various agronomic traits under normal and heat stress conditions. A total of 40% haplotypes were significantly associated with ASI, EH, EPO and GY under heat stress. The present study illustrated that both SNP-based and haplotype-based association mapping are robust for deciphering the genetic architecture of heat stress using high density SNPs. Comprehensively, 9 and 7 candidate genes, respectively, were identified from SNP-based and haplotype-based association mapping. A total of 38% significantly associated haplotypes comprising one or more SNPs were identified by Contreras-Soto et al. [48] while Chen et al. [35] identified 15 haplotypes significantly associated with *Fusarium* ear rot (FER) resistance and each SNP had comparatively small additive effects on disease resistance explaining 1–4% of phenotypic variation. Similarly, Rashid et al. [49] found 12 SNPs and four haplotype blocks associated with sorghum downy mildew in maize and one of the SNP S2_6154311 present on chromosome 2 contributed 26.7% of the trait variation. Likewise, in the present study we identified 25 haplotypes for GY and ASI under normal conditions as compared to 12 and 17 haplotypes for GY and ASI, respectively, under heat stress. Carlos et al. [30] identified 40 haplotype blocks that were significantly associated with leaf angle and one of the haplotypes hapLA4.04 accounted up to 29% of the phenotypic variation which was consistent over two seasons.

A SNP, S10_1,905,274 and three blocks (Hap_10.4, Hap_10.3 and Hap_10.2) present on chromosome 10, was co-localized with GY, PH and EH under normal conditions and this SNP was confined to gene model, GRMZM2G104620. Almeida et al. [50] reported that chromosome 7 at 123.61 to 132.68 Mb and chromosome 3 at 169.75 to 178.23 Mb genomic regions have been associated with drought tolerance or drought adaptation for grain yield and ASI under well-watered and drought stress conditions as these genomic regions harbor 5 and 6 meta QTLs [50]. In the present study, the SNPs found on chromosomes 3 and 7 did lies within the given base pairs. Hence, it needs further thorough study and validation of the associated SNPs to be utilized in the breeding programs for developing heat tolerance cultivar. We were able to identify the marker trait associations (MTAs) under heat stress and to pinpoint the involvement of different chromosomal regions due to normal and heat-stressed conditions.

Some significant SNPs and haplotype blocks were found within different gene models such as 40S ribosomal protein subunit, universal stress protein domain containing protein, expressed protein, protein kinase family protein, GRAS family transcription factor containing protein, GATA zinc finger domain containing protein, SAP domain containing protein, outer membrane protein OMP85 family, pentatricopeptide repeat super family protein, Pheophorbide a oxygenase chloroplast precursor and U-box domain-containing protein. These genes are involved in various cellular and metabolic processes in crops. One of the candidate genes, GRMZM2G887068, detected under heat stress for EH and EPO has a physiological role associated with ion, scavenging hypoxia responses, cellular mobility and regulation of cell growth and development, and role in resistance to multiple stresses [51]. Other predicted candidate genes were associated with different traits under normal conditions. The candidate genes significantly associated with GY + other traits were GRMZM2G31624, GRMZM2G104620, AC198366.3_FGT004 & GRMZM2G317287, GRMZM2G057557 and GRMZM2G418432 encoding U-box domain-containing protein, expressed protein, GRAS family transcription factor containing protein expressed, outer membrane protein OMP85 family putative expressed and protein kinase family protein, respectively (Additional file 5: Table S2). These genes are involved in regulation of plant growth and development and protein translocation. The respective genes could be targeted in maize breeding programs for enhancement of multiple traits simultaneously.

## Conclusions

Significant SNPs and haplotype blocks found associated with yield related traits, directly or indirectly related to heat stress mechanism, which would help in selection of donor lines or lines with favorable alleles for several traits. Highest number of SNPs were identified on the chromosome 8 (47 SNPs) followed by chromosome 10 (46 SNPs), chromosome 1 (36 SNPs), chromosome 3 (25 SNPs), chromosome 7 (19 SNPs) and chromosome 6 (10 SNPs) for the various agronomic traits under normal and heat stress conditions. Before introducing to breeding pipelines, significant SNPs and haplotype blocks need further validation and it will be great help for understanding of complex genetic architecture traits under heat stress.

## Methods

### Plant materials

A total of six hundred and sixty-two doubled haploid (DH) lines test crosses derived with a heat susceptible tester line (CML-474) were received from CIMMYT-Asia Regional Maize Program, Hyderabad, India. CML474 belongs to heterotic group B that is why it was used as male. The evaluation of the test cross with susceptible tester always reflects the female line effect. The DH lines were derived from nine bi-parental crosses involving elite plant material of the working germplasm from CIMMYT-Asia. The female parental lines involved in the cross have been identified as potential heat tolerant elite lines in previous experiments, while the male parental lines are susceptible but elite lines of the program. The DH lines are being maintained with CIMMYT-Asia maize breeding program hub based out of Hyderabad, India, which are available for research on request. A detailed description of these lines has been provided in Additional file 6: Table S3.

### Phenotypic evaluation of the association mapping panel

The DH lines were phenotypically evaluated at Punjab Agricultural University, Ludhiana (Punjab), a hot spot location for heat stress in India. Ludhiana is geographically situated at 30.91°N latitude and 75.85°E longitude and at an altitude of 256 m above sea level. Field-based assessment of panel was done during spring seasons (February–May) 2016 and 2017. The experiment was conducted in Alpha Lattice design with two replications each under normal sown and late sown conditions. Each entry was represented by single row of 3 m length with a spacing of 65 cm between rows and 20 cm between plants. The late sowing trial was delayed (1st week of March) to ensure maximal heat stress during flowering and grain formation during April/May months. Control or normal sowing was done on 1st week of February with same set of lines. As per meteorological data, late sown genotypes (natural heat stress) were coincided with high temperature during plant developmental and pollination stages (Additional file 7: Table S4).

### Observation recorded

During the growing season, data was recorded for grain yield contributing traits and secondary traits. Days to 50% anthesis (AD) and silking (SD) were recorded as the date when half of the plants extruded the first anthers and silks in the plot. Anthesis-silking interval (ASI) was calculated as the difference between AD and SD. Leaf firing and tassel blast was recorded as the percentage of plants in a plot with leaf firing and tassel blast symptoms. Plant height (PH) was measured from the soil surface to the base of the tassel (excluding tassel length) and ear height (EH) was measured from the soil surface to the base of the ear. Ear position (EPO) was calculated as the ratio of plant height and ear height. Grain yield (GY) was recorded in terms of ear weight per plot immediately after crop harvest at 12.5% grain moisture content and 80% shelling percentage and transformed to tons per hectare.

### Phenotypic data analysis

Genotypic (σ^2^Gen), error (σ^2^e), and genotype × environment (σ^2^Gen × Env) variance components for the phenotypic data were estimated from analysis of variance (ANOVA) and other statistical analysis were performed using standard procedures with R (METAR) [52]. The linear model used for the analysis is

$$ {\mathrm{Y}}_{\mathrm{i}\mathrm{jkl}}=\mu +{\mathrm{Env}}_{\mathrm{i}}+{\mathrm{Rep}}_{\mathrm{j}}\left({\mathrm{Env}}_{\mathrm{i}}\right)+{\mathrm{Block}}_{\mathrm{k}}\left({\mathrm{Env}}_{\mathrm{i}}\mathrm{Rep}\right)+{\mathrm{Gen}}_{\mathrm{l}}+{\mathrm{Env}}_{\mathrm{i}}\times {\mathrm{Gen}}_{\mathrm{l}}+{\varepsilon}_{\mathrm{i}\mathrm{jkl}} $$

where Y_ijkl_ represents the trait, μ is the mean effect, Env_i_ represents the effect of ith environment Rep_j_(Env_i_) is the effect of the jth replicate in environment i, Block_k_(Env_i_ Rep_j_) is the effect of kth incomplete block within the jth replicate in ith environment, Gen_l_ is the effect of the kth genotype, Env_i_ × Gen_l_ represents the genotype × environment effects and ε_ijkl_ is the residual associated with the ith environment, jth replication, kth incomplete block and lth genotype, which is assumed to be normally and independently distributed, with mean zero and homocedastic variance. The best linear unbiased predictors and variance components were estimated considering all the effects in the model as random. Broad-sense heritability (H^2^) of the traits was estimated using the formula [53]:

$$ {\mathrm{H}}^2={\sigma}^2\mathrm{Gen}/\left({\sigma}^2\mathrm{Gen}+{\sigma}^2\mathrm{Gen}\mathrm{xEnv}/l+{\sigma}^2\mathrm{e}/l\ \mathrm{r}\right) $$

Where, σ^2^ Gen = genotypic variance, σ^2^ Gen × Env = genotype × environment variance, σ^2^ e = error variance, *l* = number of environments and r = number of replications.

### Genotyping

DNA was extracted from leaf samples of three to 4 weeks old seedlings using the standard CIMMYT laboratory protocol [54]. The nine DH populations under study were genotyped using Genotyping-by-Sequencing (GBS) approach at the Institute for Genomic Diversity, Cornell University, Ithaca, NY, USA [55]. Genomic DNA was restricted with ApeK1 and GBS libraries were generated in 96-plex which were further sequenced on Illumina HiSeq 2000. TASSEL-GBS pipeline was used for SNP calling with B73 as the reference genome [56]. Physical co-ordinates of all SNPs were ascertained from B73 AGPV2 reference genome. The genotypic data of approximately 22,000 maize samples comprises 955,690 SNPs across all the chromosomes is available at https://hdl.handle.net/11529/10548550 in the imputed GBS SNP dataset. A total of 187,000 SNPs for 662 DHs from the above mentioned dataset was used for GWAS which met the filtering criteria of Call Rate (CR) ≥0.7 and Minor Allele Frequency (MAF) ≥0.1.

### GWAS analysis

The principal component analysis (PCA) was computed using the R package GAPIT (Genome Association and Prediction Integrated Tool) to estimate the number of subpopulations within the DH lines [57]. For each trait, BLUPs values of 643 DHs under normal and heat stress conditions across the years were used for genome-wide association studies (GWAS) (Additional file 8: Table S5 and Additional file 9: Table S6). The traits which had heritability equal to 0.2 or greater were used for further GWAS analysis as it was considered as moderate heritability for traits under abiotic stress management. Mixed linear model (MLM) was implemented for detecting significantly associated SNPs using R package GAPIT [57]. The false associations were corrected using both principal components (PCs) and kinship matrix for high precision. The combination of all PCs has greater power to detect the genetic variants (SNPs) with more robustness. The quantile-quantile (Q-Q)-plot and Manhattan plot were generated with customized R scripts to identify the SNP-trait associations [57]. Significant traits-SNPs associations were selected based on an arbitrary but high threshold cut-off *P* value (≤10^− 5^) [58–60]. The location of each associated SNP within gene was attained from the Maize GDB genome browser (www.maizegdb.org) and functional gene annotations were retrieved from http://ensembl.gramene.org/*Zea_mays*.

### Haplotypes trend regression

Haplotype frequency estimation was done among all the SNPs within the bottom 0.1 percentile of the distribution in the GWAS [60] for all traits, using 50 Expectation Maximization (EM) algorithm with EM convergence tolerance of 0.0001 and a frequency threshold of 0.01 [61] as implemented in SVS V_8.6.0 (Golden Helix, Inc., Bozeman, MT, www.goldenhelix.com) [49]. Haplotype blocks were identified to minimize historical recombinations based on the block-defining algorithm [62]. Stepwise regression of the trait phenotype with the haplotypes and SNPs was performed with forward elimination and a *P*-value cut-off of 0.001 [48]. A Bonferroni correction P value cut off of less than or equal to 0.05 was used to define significant association.

## Supplementary Information

**Additional file 1: Figure S1.** Frequency distribution of 662 doubled haploid (DH) lines for various traits under normal conditions.GY = Grain yield (A), AD = Days to 50% anthesis (B), SD = Days to 50% silking (C), PH = Plant height (D), EH = Ear height (E) and EPO = Ear position (F).

**Additional file 2: Figure S2.** Frequency distribution of 662 doubled haploid (DH) lines for various traits under heat stress conditions.GY = Grain yield (A), ASI = Anthesis-silking interval (B), EH = Ear height (C) and EPO = Ear position (D).

**Additional file 3: Figure S3.** Principle components analysis (PCA) showing the first four principle components using the genotyping by sequencing data.

**Additional file 4: Table S1.** Significant marker-trait associations for across environments under normal and heat stress conditions.

**Additional file 5: Table S2.** Summary of the candidate genes associated with the different traits.

**Additional file 6: Table S3.** List of doubled haploid (DH) lines used in the present investigation for evaluation under normal and heat stress conditions.

**Additional file 7: Table S4.** Minimum and maximum temperature at the time of sowing to crop maturity under normal and late sown conditions during 2016 and 2017 years.

**Additional file 8: Table S5.** Best linear unbiased prediction value (BLUPs) for DH population under normal condition across two years.

**Additional file 9: Table S6.** Best linear unbiased prediction value (BLUPs) for DH population under heat stress conditions across two years.

## Abbreviations

ASI: Anthesis-silking interval
AD: Days to 50% anthesis
BLUP: Best Linear Unbiased Prediction
CT: Canopy temperature
CIMMYT: International Maize and Wheat Improvement Centre
Env: Environment
EH: Ear height
EPO: Ear position
Gen: Genotype
GY: Grain yield
GWAS: Genome-wide association study
H^2^: Broad-sense heritability
HTR: Haplotype Trend Regression
METAR: Multi-Environment Trail Analysis with R
MLM: Mixed Linear Model
PH: Plant height
PCs: Principal components
PCA: Principle component analysis
QTL: Quantative trait loci
PV: Percentage of phenotypic variation explained by the associated SNPs
SNPs: Single nucleotide polymorphisms.

## Declarations

### Availability of the data and materials

All supporting data can be found within the manuscript and its additional files. The GBS datasets analysed during the current study are available at https://hdl.handle.net/11529/10548550.
