# Supplementary figures and images for: Genome wide association mapping for heat tolerance in sub-tropical maize
Source: BMC Genomics. 2021 Mar 4;22:154. doi: 10.1186/s12864-021-07463-y (PMC7934507; doi:10.1186/s12864-021-07463-y)

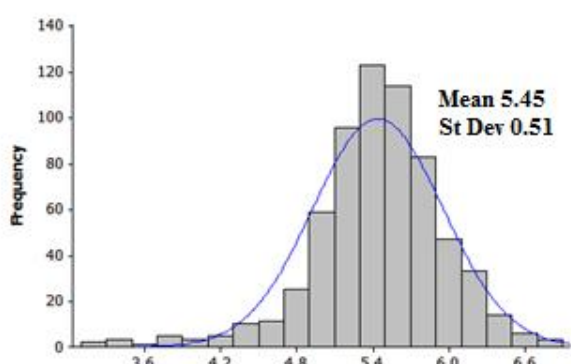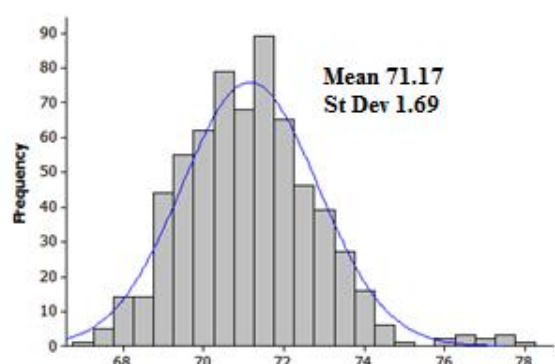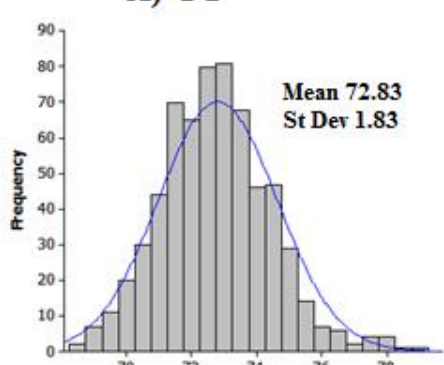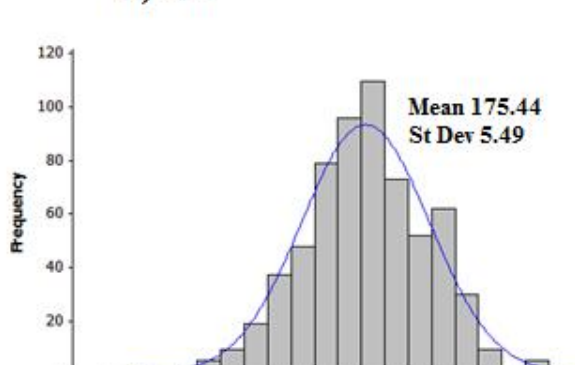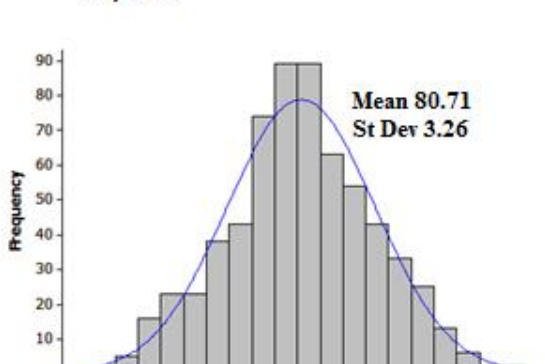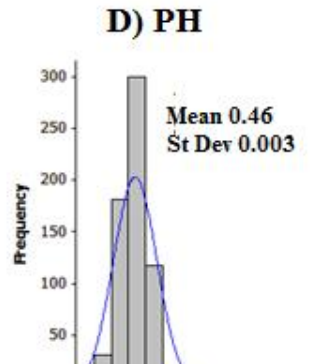

Supplement: Supplementary file 1 — Additional file 1: Figure S1. Frequency distribution of 662 doubled haploid (DH) lines for various traits under normal conditions.GY = Grain yield (A), AD = Days to 50% anthesis (B), SD = Days to 50% silking (C), PH = Plant height (D), EH = Ear height (E) and EPO = Ear position (F). [file 12864_2021_7463_MOESM1_ESM.pdf]

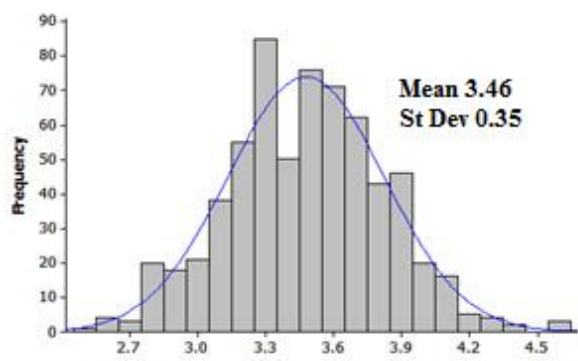

**A) GY**

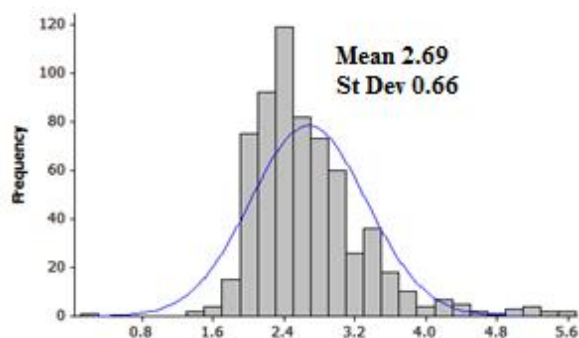

**B) ASI**

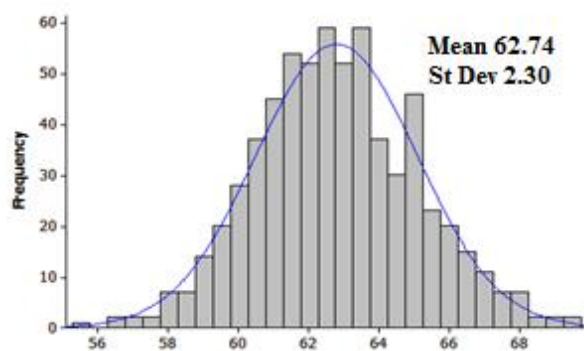

**C) EH**

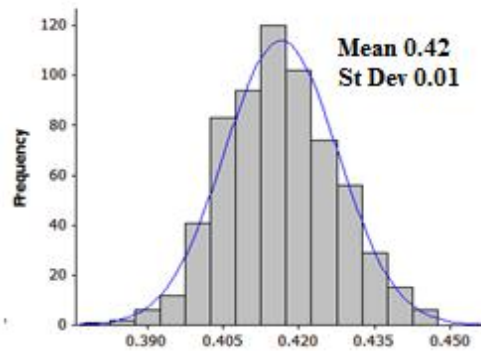

**D) EPO**

Supplement: Supplementary file 2 — Additional file 2: Figure S2. Frequency distribution of 662 doubled haploid (DH) lines for various traits under heat stress conditions.GY = Grain yield (A), ASI = Anthesis-silking interval (B), EH = Ear height (C) and EPO = Ear position (D). [file 12864_2021_7463_MOESM2_ESM.pdf]

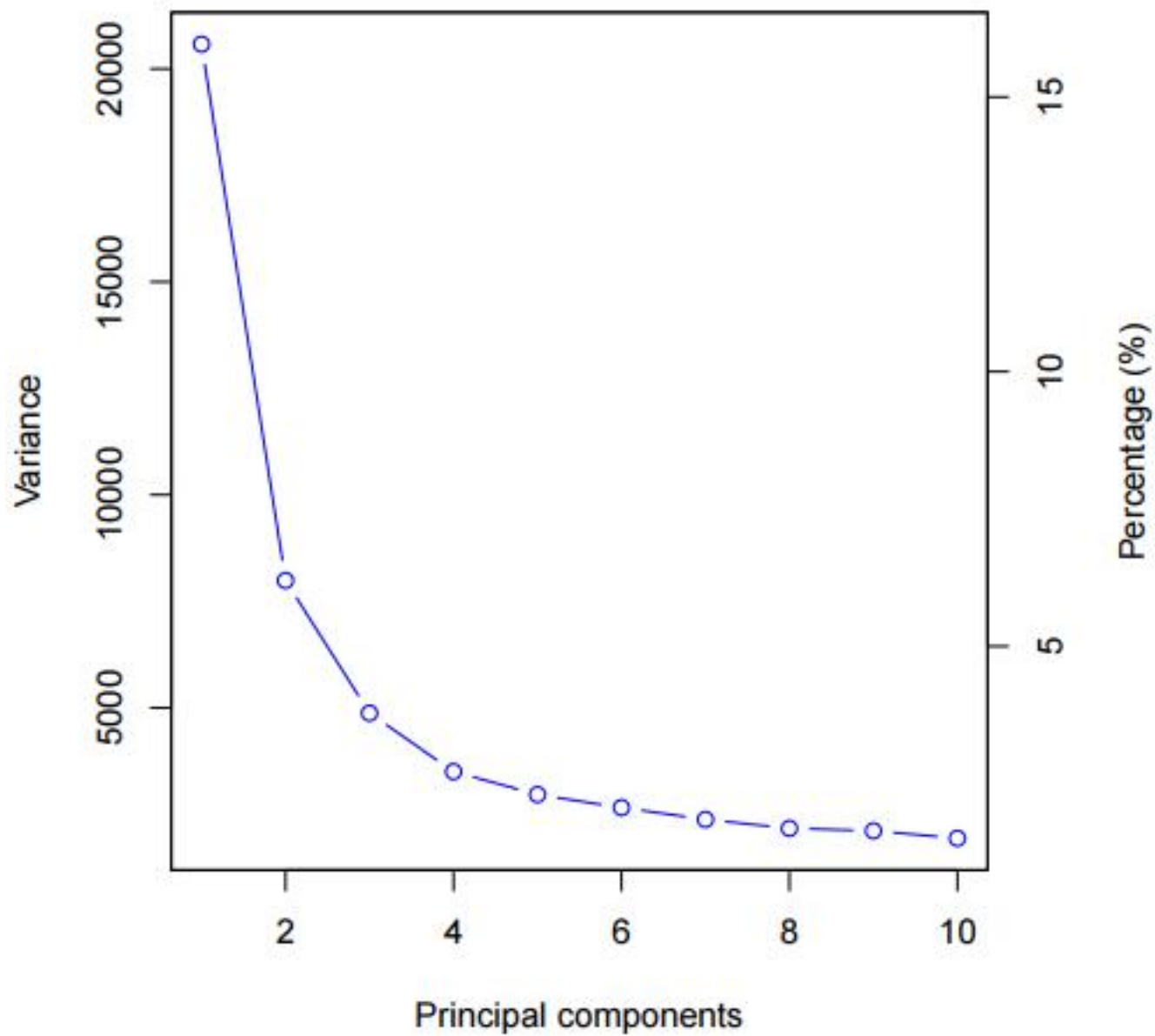

Supplement: Supplementary file 3 — Additional file 3: Figure S3. Principle components analysis (PCA) showing the first four principle components using the genotyping by sequencing data. [file 12864_2021_7463_MOESM3_ESM.pdf]
