# Supplementary material for: Genome wide association mapping for heat tolerance in sub-tropical maize
Source: BMC Genomics. 2021 Mar 4;22:154. doi: 10.1186/s12864-021-07463-y (PMC7934507; doi:10.1186/s12864-021-07463-y)
Supplement: Supplementary file 4 — Additional file 4: Table S1. Significant marker-trait associations for across environments under normal and heat stress conditions. [file 12864_2021_7463_MOESM4_ESM.pdf]

Table S1. Significant marker-trait associations for across environment under normal and heat stress conditions.

Environment	Trait	SNP	Chromosome	Position (bp)	P-value	MAF	R Squared	SNP effect
Normal	AD	S6_156527428	6	1.57E+08	1.46E-05	0.214619	0.457755	0.07656
		S6_156527431	6	1.57E+08	1.46E-05	0.214619	0.457755	0.010515
		S6_156527432	6	1.57E+08	1.46E-05	0.214619	0.457755	0.010515
		S1_295456768	1	2.95E+08	2.11E-05	0.33437	0.457124	0.046497
		S1_4752039	1	4752039	2.11E-05	0.158631	0.457121	0.040603
		S10_144031682	10	1.44E+08	2.58E-05	0.225505	0.456775	-0.01051
		S6_156527456	6	1.57E+08	2.87E-05	0.220062	0.456597	0.010515
		S6_156527380	6	1.57E+08	3.36E-05	0.187403	0.456323	0.07656
		S8_137928804	8	1.38E+08	5.74E-05	0.11353	0.455411	0.04121
		S6_121209677	6	1.21E+08	6.14E-05	0.158631	0.455295	0.101046
		S10_7132845	10	7132845	6.74E-05	0.103421	0.455138	0.129472
		S1_211451569	1	2.11E+08	8.09E-05	0.171073	0.454827	-0.03107
		S10_3643760	10	3643760	8.51E-05	0.37014	0.454742	0.044623
		S3_153048425	3	1.53E+08	8.64E-05	0.183515	0.454715	0.041102
		S1_293631936	1	2.94E+08	9.37E-05	0.440124	0.454578	0.166124
		S1_293631937	1	2.94E+08	9.37E-05	0.440124	0.454578	-0.04003
		S1_228565627	1	2.29E+08	9.53E-05	0.267496	0.454549	0.046247
	ASI	S6_153198366	6	1.53E+08	6.52E-07	0.316485	0.218875	-0.00953
		S10_8852411	10	8852411	1.03E-06	0.220062	0.21772	0.0209
		S10_9473175	10	9473175	1.08E-06	0.233281	0.217577	-0.01494
		S10_9473057	10	9473057	1.50E-06	0.192846	0.216758	0.045499
		S10_2007234	10	2007234	1.57E-06	0.101866	0.216642	0.03519
		S6_152509426	6	1.53E+08	3.63E-06	0.405132	0.21451	0.0253
		S6_152250498	6	1.52E+08	5.18E-06	0.209953	0.213612	-0.00709
		S10_2044119	10	2044119	6.31E-06	0.11042	0.213111	0.000762
		S1_240038346	1	2.4E+08	6.53E-06	0.134526	0.213026	-0.07128
		S10_8273843	10	8273843	7.76E-06	0.489891	0.212592	-0.03344
		S6_153260968	6	1.53E+08	7.94E-06	0.225505	0.212534	0.013634
		S6_153261154	6	1.53E+08	9.34E-06	0.216952	0.212126	-0.03539
		S6_154154395	6	1.54E+08	9.50E-06	0.22084	0.212082	-0.04418
		S8_16321230	8	16321230	9.86E-06	0.281493	0.211988	0.041746
		S10_9473157	10	9473157	1.25E-05	0.139191	0.211399	-0.01494

		S6_153260951	6	1.53E+08	1.26E-05	0.224728	0.211379	-0.07136
		S1_24602336	1	24602336	1.59E-05	0.116641	0.21079	-0.05347
		S1_5719699	1	5719699	1.94E-05	0.115863	0.21029	0.03265
		S6_153261312	6	1.53E+08	1.95E-05	0.37014	0.210282	-0.0224
		S6_157167897	6	1.57E+08	2.21E-05	0.11042	0.209961	0.002398
		S10_132091328	10	1.32E+08	2.26E-05	0.16563	0.209914	-0.01314
		S6_156881216	6	1.57E+08	2.36E-05	0.209953	0.209804	0.016465
		S1_255325030	1	2.55E+08	2.40E-05	0.171073	0.209758	-0.05052
		S8_16770530	8	16770530	2.69E-05	0.212286	0.209478	0.063968
		S6_156527416	6	1.57E+08	2.75E-05	0.199844	0.209418	0.046857
	EH	S8_170952700	8	1.71E+08	5.53E-06	0.450233	0.237893	-0.32207
		S10_1888234	10	1888234	6.40E-06	0.448678	0.237534	-0.14973
		S7_10559998	7	10559998	2.20E-05	0.368585	0.234528	0.110954
		S10_137166772	10	1.37E+08	3.63E-05	0.214619	0.233326	-0.10091
		S2_59729573	2	59729573	4.49E-05	0.440124	0.232813	-0.42962
		S2_59729575	2	59729575	4.49E-05	0.440124	0.232813	0.345127
		S2_59729611	2	59729611	4.49E-05	0.440124	0.232813	0.334602
		S8_133184306	8	1.33E+08	5.51E-05	0.306376	0.232317	-0.05718
		S10_130232613	10	1.3E+08	6.94E-05	0.116641	0.231765	-0.25372
		S10_137166781	10	1.37E+08	7.62E-05	0.217729	0.231539	-0.10091
		S10_1280109	10	1280109	8.71E-05	0.238725	0.231222	-0.21959
		S10_1905273	10	1905273	9.21E-05	0.461897	0.231087	-0.56601
		S10_1905274	10	1905274	9.21E-05	0.461897	0.231087	-0.35828
		S8_170952712	8	1.71E+08	9.70E-05	0.465008	0.230963	0.164872
	EPO	S5_214350949	5	2.14E+08	7.67E-07	0.175739	0.058531	1.13E-05
		S1_23065763	1	23065763	8.92E-06	0.497667	0.051038	0.000175
		S9_147503575	9	1.48E+08	1.47E-05	0.143857	0.049534	8.42E-05
		S2_226366816	2	2.26E+08	2.90E-05	0.157076	0.047484	-0.00014
		S3_159381162	3	1.59E+08	3.80E-05	0.140747	0.04667	0.000155
		S3_159676137	3	1.6E+08	5.00E-05	0.357698	0.045853	-0.00022
		S3_159676248	3	1.6E+08	7.02E-05	0.316485	0.044839	4.73E-05
		S3_159676324	3	1.6E+08	9.23E-05	0.332037	0.044021	-0.00011
		S2_220143014	2	2.2E+08	9.38E-05	0.166407	0.043974	-5.00E-05
		S2_220143015	2	2.2E+08	9.38E-05	0.166407	0.043974	-5.00E-05
		S8_168952873	8	1.69E+08	9.62E-05	0.186625	0.0439	-1.50E-05

	GY	S10_2256100	10	2256100	1.95E-07	0.191291	0.232308	0.013712
		S10_5642063	10	5642063	3.74E-07	0.117418	0.230653	-0.0375
		S10_7132845	10	7132845	7.03E-07	0.103421	0.229059	0.001808
		S10_10826645	10	10826645	1.12E-06	0.11042	0.227888	0.015764
		S10_1148841	10	1148841	1.75E-06	0.354588	0.22677	-0.04941
		S10_5528080	10	5528080	2.04E-06	0.177294	0.226382	-0.03823
		S10_5528122	10	5528122	2.04E-06	0.177294	0.226382	-0.03823
		S3_226971587	3	2.27E+08	4.20E-06	0.175739	0.224573	-0.00912
		S10_2988861	10	2988861	4.22E-06	0.111975	0.224562	0.020573
		S10_2986806	10	2986806	4.98E-06	0.125194	0.224151	-0.02609
		S10_2142412	10	2142412	5.09E-06	0.478227	0.224093	-0.01268
		S10_1905273	10	1905273	6.14E-06	0.461897	0.22363	-0.04119
		S10_1905274	10	1905274	6.14E-06	0.461897	0.22363	-0.03467
		S10_8844033	10	8844033	8.16E-06	0.290047	0.222922	0.012039
		S10_1902657	10	1902657	9.51E-06	0.265941	0.222541	0.001654
		S10_1888234	10	1888234	1.09E-05	0.448678	0.222205	-0.04032
		S10_2142394	10	2142394	1.75E-05	0.468118	0.221037	0.034565
		S10_9399962	10	9399962	1.85E-05	0.150855	0.220894	-0.04989
		S7_11667743	7	11667743	2.44E-05	0.247278	0.220216	-0.01689
		S10_9399963	10	9399963	2.64E-05	0.153188	0.220016	-0.02852
		S7_20192131	7	20192131	2.86E-05	0.216174	0.219826	0.001113
		S10_6860384	10	6860384	3.01E-05	0.31804	0.219696	-0.04302
		S1_187641539	1	1.88E+08	3.68E-05	0.418351	0.219202	-0.0427
		S10_1883817	10	1883817	3.83E-05	0.492224	0.219104	-0.0115
		S10_5533177	10	5533177	3.95E-05	0.18196	0.219026	-0.052
	PH	S10_1905273	10	1905273	4.14E-07	0.461897	0.270389	-0.74036
		S10_1905274	10	1905274	4.14E-07	0.461897	0.270389	-0.41266
		S10_1714669	10	1714669	4.98E-07	0.186625	0.269947	0.546315
		S10_1883817	10	1883817	5.44E-06	0.492224	0.26426	-0.08339
		S10_1727328	10	1727328	1.75E-05	0.234837	0.261511	-0.21361
		S10_2142382	10	2142382	2.65E-05	0.342146	0.260543	0.513749
		S10_2142391	10	2142391	2.65E-05	0.342146	0.260543	0.301947
		S10_2142404	10	2142404	2.65E-05	0.342146	0.260543	0.680257
		S10_1905237	10	1905237	3.74E-05	0.372473	0.259742	0.297045
		S8_107194228	8	1.07E+08	4.22E-05	0.262053	0.259459	-0.07321

		S8_112150133	8	1.12E+08	5.30E-05	0.430793	0.258928	0.114587
		S1_298963146	1	2.99E+08	5.74E-05	0.138414	0.258743	0.032125
		S10_1148841	10	1148841	5.81E-05	0.354588	0.258716	-0.41877
		S10_3192709	10	3192709	6.22E-05	0.276827	0.258557	0.096102
		S2_18211312	2	18211312	6.37E-05	0.452566	0.258501	-0.02502
		S10_6373070	10	6373070	7.19E-05	0.131415	0.258223	0.379466
		S2_18210872	2	18210872	8.21E-05	0.360031	0.257917	-0.20932
		S10_2402911	10	2402911	9.27E-05	0.450233	0.257636	-0.59559
		S2_16940324	2	16940324	9.69E-05	0.302488	0.257533	-0.49754
	SD	S10_10826645	10	10826645	6.22E-06	0.11042	0.392132	0.108606
		S7_142777920	7	1.43E+08	6.40E-06	0.18818	0.392076	-0.1293
		S10_2133486	10	2133486	1.46E-05	0.241835	0.390473	-0.17677
		S10_8931708	10	8931708	3.60E-05	0.268274	0.388735	0.027699
		S6_156527428	6	1.57E+08	4.69E-05	0.214619	0.388226	0.077691
		S6_156527431	6	1.57E+08	4.69E-05	0.214619	0.388226	0.006146
		S6_156527432	6	1.57E+08	4.69E-05	0.214619	0.388226	0.006146
		S10_8852411	10	8852411	5.75E-05	0.220062	0.387837	-0.05648
		S10_7132845	10	7132845	5.83E-05	0.103421	0.387811	0.065393
		S10_10621204	10	10621204	5.99E-05	0.153966	0.387758	0.024369
		S1_228565627	1	2.29E+08	6.65E-05	0.267496	0.387558	0.008173
		S10_7440298	10	7440298	6.89E-05	0.122084	0.387489	0.080236
		S1_4752039	1	4752039	7.20E-05	0.158631	0.387405	-0.01541
		S4_239683229	4	2.4E+08	7.54E-05	0.151633	0.387318	-0.00465
		S6_8680494	6	8680494	7.70E-05	0.22395	0.387277	-0.02472
		S3_155735931	3	1.56E+08	8.02E-05	0.475894	0.387199	0.074499
		S10_9473175	10	9473175	8.14E-05	0.233281	0.387172	-0.08
		S6_156527380	6	1.57E+08	8.56E-05	0.187403	0.387075	0.077691
		S1_103418790	1	1.03E+08	9.50E-05	0.141524	0.386877	0.334394
Heat stress	ASI	S2_217334975	2	2.17E+08	4.12E-06	0.251944	0.285324	0.004452
		S1_207528245	1	2.08E+08	6.19E-06	0.13297	0.284393	0.042094
		S6_97382439	6	97382439	8.39E-06	0.11042	0.283695	0.025702
		S6_96916118	6	96916118	1.30E-05	0.134526	0.282687	-0.05079
		S1_235975064	1	2.36E+08	1.92E-05	0.183515	0.281802	0.016012
		S1_235909625	1	2.36E+08	2.06E-05	0.327372	0.281652	0.139625
		S1_235975061	1	2.36E+08	3.97E-05	0.181182	0.280159	-0.01241

		S1_235909649	1	2.36E+08	4.05E-05	0.326594	0.280112	0.142785
		S1_235975170	1	2.36E+08	4.36E-05	0.18507	0.279945	-0.08939
		S1_206876087	1	2.07E+08	4.92E-05	0.416796	0.279674	0.008309
		S1_206876091	1	2.07E+08	4.92E-05	0.416796	0.279674	-0.00021
		S1_235909741	1	2.36E+08	7.26E-05	0.323484	0.278797	0.013156
		S3_165852541	3	1.66E+08	7.39E-05	0.115863	0.278758	-0.00964
		S1_235974531	1	2.36E+08	7.70E-05	0.177294	0.278665	0.014534
		S1_286981761	1	2.87E+08	9.25E-05	0.118974	0.278253	0.072579
		S1_240893351	1	2.41E+08	9.85E-05	0.247278	0.278113	0.014732
		S6_102548419	6	1.03E+08	9.90E-05	0.129082	0.278101	-0.05088
	EH	S8_75642171	8	75642171	7.66E-06	0.143857	0.26265	0.070232
		S8_79188751	8	79188751	1.34E-05	0.186625	0.261341	0.106305
		S8_149221776	8	1.49E+08	1.97E-05	0.286936	0.260432	0.038137
		S10_1498474	10	1498474	2.05E-05	0.236392	0.260333	-0.08174
		S8_79150473	8	79150473	3.43E-05	0.186625	0.259135	0.146416
		S8_80220477	8	80220477	3.90E-05	0.137636	0.258837	0.048209
		S8_79148206	8	79148206	4.16E-05	0.153188	0.258685	0.132934
		S8_82493687	8	82493687	6.81E-05	0.157076	0.257539	0.120869
		S8_100873013	8	1.01E+08	8.19E-05	0.304821	0.257112	0.348913
		S8_79150233	8	79150233	8.66E-05	0.136081	0.256984	-0.13337
		S3_161368749	3	1.61E+08	8.97E-05	0.262053	0.256901	0.088714
		S8_79148192	8	79148192	9.17E-05	0.142302	0.25685	-0.07033
		S8_66406765	8	66406765	9.53E-05	0.154743	0.256763	-0.20475
		S8_92967148	8	92967148	9.75E-05	0.356921	0.256708	0.111387
	EPO	S8_131584023	8	1.32E+08	1.81E-05	0.343701	0.219512	0.000178
		S8_131584041	8	1.32E+08	2.20E-05	0.342924	0.219032	0.000442
		S8_131584186	8	1.32E+08	8.79E-05	0.33437	0.215628	-0.00052
	GY	S1_3000739	1	3000739	1.18E-05	0.281493	0.186533	0.012899
		S6_154325003	6	1.54E+08	1.89E-05	0.360031	0.185311	-0.00459
		S1_2753089	1	2753089	3.45E-05	0.355365	0.183762	0.023675
		S6_150736678	6	1.51E+08	3.80E-05	0.210731	0.183514	0.018685
		S1_7714403	1	7714403	4.30E-05	0.388802	0.183199	0.012604
		S3_227087108	3	2.27E+08	4.57E-05	0.307932	0.183042	0.012276
		S7_120765488	7	1.21E+08	5.15E-05	0.20451	0.182738	0.029245
		S7_120765490	7	1.21E+08	5.38E-05	0.205288	0.182623	0.028151

		S7_120765491	7	1.21E+08	5.38E-05	0.205288	0.182623	-0.02924
		S6_155634322	6	1.56E+08	8.64E-05	0.477449	0.181414	-0.0006
		S10_22867964	10	22867964	8.80E-05	0.117418	0.181367	0.023988
		S10_22867970	10	22867970	8.80E-05	0.117418	0.181367	0.018633

P-value= Significant threshold level, MAF= Minor allele frequency, R squared= Proportion of total variance explained by SNP, AD= Days to 50% anthesis, SD= Days to 50% silking, ASI= Anthesis-silking interval, PH= Plant height, EH= Ear height, EPO= Ear position and GY= Grain yield.
