# Supplementary material for: Genome wide association mapping for heat tolerance in sub-tropical maize
Source: BMC Genomics. 2021 Mar 4;22:154. doi: 10.1186/s12864-021-07463-y (PMC7934507; doi:10.1186/s12864-021-07463-y)
Supplement: Supplementary file 5 — Additional file 5: Table S2. Summary of the candidate genes associated with the different traits. [file 12864_2021_7463_MOESM5_ESM.pdf]

Table S2. Summary of the candidate genes associated with the different traits.

Trait	Gene Model	Description
AD + SD	GRMZM5G877815	40S ribosomal protein 527a putative expressed
AD + SD + GY	GRMZM2G031624	U-box domain-containing protein putative expressed
ASI + SD	GRMZM2G438176	SAP domain containing protein expressed
ASI + SD	GRMZM2G048850	GATA zinc finger domain containing protein expressed
EH + GY	AC198366.3_FGT004	GRAS family transcription factor containing protein expressed
EH + GY + PH	GRMZM2G104620	Expressed protein
GY + SD	GRMZM2G418432	protein kinase family protein putative expressed
GY + PH	GRMZM2G057557	outer membrane protein OMP85 family putative expressed
GY + PH	GRMZM2G317287	GRAS family transcription factor containing protein expressed
GY + ASI + SD	GRMZM2G583593	Nil
AD + SD	GRMZM2G056594	Pentatricopeptide repeat (PPR) superfamily protein
AD + SD	GRMZM2G018484	Pheophorbide a oxygenase chloroplast precursor
AD + SD	GRMZM2G109144	Expressed protein
EH + EPO	GRMZM2G379128	Pentatricopeptide repeat (PPR) superfamily protein
EH + EPO	GRMZM2G887068	Universal stress protein domain containing protein putative expressed

AD= Days to 50% anthesis, SD= Days to 50% silking, ASI= Anthesis-silking interval, PH= Plant height, EH= Ear height, EPO= Ear position and GY= Grain yield.
