# Supplementary material for: Genome wide association mapping for heat tolerance in sub-tropical maize
Source: BMC Genomics. 2021 Mar 4;22:154. doi: 10.1186/s12864-021-07463-y (PMC7934507; doi:10.1186/s12864-021-07463-y)
Supplement: Supplementary file 6 — Additional file 6: Table S3. List of doubled haploid (DH) lines used in the present investigation for evaluation under normal and heat stress conditions. [file 12864_2021_7463_MOESM6_ESM.pdf]

Table S3. List of doubled haploid (DH) lines used in the present investigation for evaluation under normal and heat stress conditions.

S.No.	Population code	Pedigree	No. of DH lines
1	YCMLZH1393	VL109524/VL1036	62
2	YCMLZH1383887	ZL152847/ZL152840	82
3	YCMLZH111666	VL1030/VL1055	62
4	YCMLZH111497	VL108869/VL1036	54
5	YCMLZH111500	VL062605/VL1036	81
6	YCMLZH1378	VL1018114/VL1036	87
7	YCMLZH1369	VL1018146/VL1036	65
8	YCMLZH138386	VL1033/VL105611	87
9	YCMLZH1383888	VL1110201/VL1110232	82
		Total	662
