# Supplementary material for: Genome wide association mapping for heat tolerance in sub-tropical maize
Source: BMC Genomics. 2021 Mar 4;22:154. doi: 10.1186/s12864-021-07463-y (PMC7934507; doi:10.1186/s12864-021-07463-y)
Supplement: Supplementary file 7 — Additional file 7: Table S4. Minimum and maximum temperature at the time of sowing to crop maturity under normal and late sown conditions during 2016 and 2017 years. [file 12864_2021_7463_MOESM7_ESM.pdf]

**Table S4.** Minimum and maximum temperature at the time of sowing to crop maturity under normal and late sown conditions during 2016 and 2017 years.

| Months | Temperature ($^0$C) | | | |
|---|---|---|---|---|
| | Minimum | | Maximum | |
| | 2016 | 2017 | 2016 | 2017 |
| **February** | 9.0 | 9.3 | 23.0 | 23.1 |
| **March** | 14.6 | 12.5 | 28.0 | 27.2 |
| **April** | 19.6 | 20.0 | 36.6 | 36.9 |
| **May** | 24.6 | 25.1 | 39.6 | 38.8 |
| **June** | 28.5 | 26.2 | 39.8 | 36.7 |
| **July** | 26.2 | 27.5 | 33.3 | 34.6 |
