# Supplementary material for: Genome wide association mapping for heat tolerance in sub-tropical maize
Source: BMC Genomics. 2021 Mar 4;22:154. doi: 10.1186/s12864-021-07463-y (PMC7934507; doi:10.1186/s12864-021-07463-y)
Supplement: Supplementary file 8 — Additional file 8: Table S5. Best linear unbiased prediction value (BLUPs) for DH population under normal condition across two years. [file 12864_2021_7463_MOESM8_ESM.pdf]

Table S5. Best linear unbiased prediction value (BLUPs) for DH population under normal condition across two years.

Taxa	GY	AD	SD	ASI	PH	EH	EPO
DH_1_10	5.932231	70.62485	71.64782	1.142543	175.2659	81.94505	0.461457
DH_1_100	5.452453	71.68432	73.24346	1.727556	176.3487	78.67261	0.457568
DH_1_101	5.706222	72.62002	73.24017	0.948363	179.238	80.36595	0.458348
DH_1_102	6.047481	70.57505	72.13064	1.569133	174.6661	80.39221	0.459611
DH_1_103	5.412463	74.5664	76.07296	1.654788	175.5727	81.40386	0.460097
DH_1_104	6.025246	71.47671	72.85611	1.562345	173.5592	79.69903	0.458723
DH_1_106	5.769562	72.84224	74.03753	1.447202	171.2103	78.62945	0.459116
DH_1_107	6.288759	71.9887	73.63763	1.739467	182.7533	83.87202	0.45965
DH_1_109	6.031278	71.83464	73.10443	1.559916	184.4323	83.62917	0.458685
DH_1_110	6.322661	72.26112	73.44202	1.377227	172.8644	80.67169	0.461
DH_1_111	5.558935	72.14569	73.17537	1.332336	166.5004	78.47308	0.460564
DH_1_113	4.771719	70.54606	72.41871	1.813059	172.5055	80.64863	0.460711
DH_1_114	5.746334	70.57644	72.27872	1.718683	172.1251	80.40482	0.459383
DH_1_115	5.335162	73.29619	75.07242	1.795856	182.4918	84.67362	0.460465
DH_1_116	5.270043	71.89052	73.27434	1.607096	170.5554	78.40923	0.459459
DH_1_117	5.319579	72.29181	72.92364	1.134147	171.4158	77.05356	0.457131
DH_1_118	5.656044	71.94128	72.97628	1.311369	179.5254	85.06035	0.462205
DH_1_119	5.293866	71.85178	73.28605	1.501341	177.4514	84.27113	0.462132
DH_1_12	6.148306	71.42317	72.44765	1.293655	178.974	79.7631	0.457734
DH_1_121	5.998963	71.84515	73.39071	1.688441	182.9318	82.81926	0.458782
DH_1_123	5.274829	71.76582	72.95446	1.563816	176.2684	80.80589	0.459163
DH_1_124	6.250889	72.20584	73.5316	1.441724	180.7177	85.95869	0.462392
DH_1_126	6.101787	71.36486	71.98317	0.994982	191.5057	87.92537	0.460908
DH_1_127	5.272761	73.53253	74.78509	1.423748	173.3576	78.87706	0.458668
DH_1_129	6.064662	72.01953	73.36024	1.534839	184.6415	86.47756	0.462121
DH_1_13	5.942593	73.09907	74.47671	1.457794	185.4423	88.44083	0.463232
DH_1_130	5.971583	71.79138	73.05458	1.48783	184.3555	83.22166	0.458713
DH_1_131	6.434177	70.82087	72.307	1.558087	182.7163	80.98998	0.457855
DH_1_132	5.681768	70.65646	71.99653	1.550517	174.7447	78.94247	0.461193
DH_1_133	5.163023	72.70571	74.34586	1.761589	176.7488	82.95295	0.460169
DH_1_135	5.502162	73.48482	74.3515	1.093296	180.7167	79.7861	0.457081
DH_1_136	5.818952	70.50674	72.06885	1.728775	179.2757	79.96147	0.457198

Taxa	GY	AD	SD	ASI	PH	EH	EPO
DH_1_137	5.61909	71.89684	73.70548	1.888647	180.3085	82.06963	0.459298
DH_1_138	5.606732	70.39655	72.8145	2.184345	174.8526	81.76236	0.460722
DH_1_139	5.3118	72.71667	74.27129	1.723533	174.3677	77.66001	0.456943
DH_1_140	5.386292	71.09549	72.81904	1.883461	174.6558	79.2908	0.45793
DH_1_141	5.273836	72.4935	74.04327	1.734516	174.4013	78.48433	0.458017
DH_1_143	5.949337	73.38298	75.12325	1.724931	171.98	81.15135	0.460454
DH_1_144	5.952308	71.55853	73.24083	1.751015	178.0525	77.67899	0.456304
DH_1_145	5.635795	73.9747	74.75945	1.10553	181.9088	81.73546	0.458745
DH_1_146	5.785111	72.89593	73.56512	1.12247	167.066	76.77827	0.458241
DH_1_147	5.710169	73.38694	75.02739	1.791533	183.1278	82.13594	0.458977
DH_1_148	5.273978	73.51335	74.63695	1.327784	178.7311	86.61261	0.463888
DH_1_149	5.37986	72.63277	74.13443	1.590191	177.3096	81.69298	0.460805
DH_1_15	6.340524	71.29282	73.31415	2.020757	181.412	84.39005	0.460768
DH_1_150	5.583113	71.49186	73.21377	1.76627	179.1024	80.65058	0.458503
DH_1_151	6.053209	70.44892	72.25072	1.801712	182.9215	84.47331	0.460412
DH_1_152	5.296128	73.10223	73.82138	1.14179	179.1002	85.69721	0.462935
DH_1_153	6.452058	71.70321	72.31307	0.973828	189.0544	88.1023	0.461382
DH_1_155	5.274462	73.13574	74.4524	1.439572	177.4745	83.50123	0.461517
DH_1_156	5.888309	70.71846	72.27403	1.608841	179.0883	81.58473	0.458709
DH_1_157	6.125745	71.05975	71.47644	0.899412	183.1122	85.22829	0.46137
DH_1_158	5.643484	73.95219	75.16833	1.277936	179.3311	81.26827	0.459608
DH_1_159	5.855246	69.36323	70.42193	1.270503	179.8431	83.19449	0.4604
DH_1_16	5.82073	72.63572	73.8377	1.432944	183.5213	84.86989	0.461176
DH_1_160	6.302889	70.91496	72.69362	1.706857	183.1282	83.3262	0.459313
DH_1_161	5.923394	71.27126	73.01238	1.689165	185.9883	83.19851	0.458832
DH_1_164	5.401918	71.77252	73.27371	1.631811	178.2508	81.72221	0.460212
DH_1_165	6.036619	73.0967	74.76316	1.638785	182.0766	86.30743	0.462633
DH_1_166	5.750089	73.61205	74.97274	1.532772	175.9401	79.88976	0.458227
DH_1_167	5.350557	73.98188	75.58796	1.678748	181.8255	81.96842	0.458674
DH_1_168	5.810377	68.85566	70.89339	1.929202	177.2836	83.13208	0.461492
DH_1_169	5.12507	72.38412	73.64953	1.285442	182.0602	81.37922	0.458353
DH_1_17	5.135272	73.09648	74.43072	1.508935	178.3	83.0542	0.461481
DH_1_170	5.229576	70.34504	71.23836	1.17905	165.577	75.23816	0.4574
DH_1_171	5.652722	72.88115	75.07473	2.072184	176.3765	79.19839	0.45789

Taxa	GY	AD	SD	ASI	PH	EH	EPO
DH_1_172	5.145543	72.90259	73.82169	1.192387	176.1259	77.81304	0.457358
DH_1_173	5.89514	72.3927	73.42152	1.240788	176.8517	80.74414	0.460025
DH_1_174	5.415462	70.43647	72.36854	1.831978	176.9243	81.94553	0.46041
DH_1_175	5.827047	72.43284	73.85157	1.535631	175.8828	78.60202	0.457566
DH_1_176	5.536324	70.90454	72.91248	1.987851	168.0485	74.61576	0.456302
DH_1_177	5.257074	72.87201	74.07215	1.359563	175.9864	81.69812	0.460382
DH_1_178	6.763694	70.14757	71.18334	1.322172	189.7111	86.64258	0.459814
DH_1_18	5.486452	72.27926	73.18674	1.256438	176.2438	78.22354	0.456627
DH_1_180	5.307441	71.79156	72.99464	1.359876	176.0101	80.50956	0.45918
DH_1_181	5.694958	73.57106	74.34474	1.16285	181.8811	84.19748	0.460578
DH_1_182	5.407333	72.91704	74.3193	1.552004	175.7541	76.82387	0.45557
DH_1_183	5.619063	72.66921	74.44131	1.862829	183.5356	84.72657	0.460712
DH_1_184	5.643537	71.23903	73.16328	1.723412	179.9623	79.01645	0.456618
DH_1_185	6.194679	70.387	70.62719	0.774339	181.9412	84.38724	0.460721
DH_1_186	6.320689	71.44255	72.94634	1.721466	186.3934	85.63403	0.460268
DH_1_187	5.349806	72.34647	73.64924	1.424364	170.1007	75.87833	0.457314
DH_1_188	5.851628	72.1893	74.28477	2.030004	180.9399	81.87201	0.45918
DH_1_189	5.548562	70.87473	72.25535	1.459974	179.7393	81.29944	0.45808
DH_1_19	5.854915	73.89858	74.65866	1.106689	188.1069	87.82625	0.461677
DH_1_190	5.724209	70.5996	71.89501	1.465479	173.6741	81.73025	0.461367
DH_1_191	5.743307	73.49983	74.82394	1.565068	179.5995	84.86347	0.461995
DH_1_192	5.720862	70.40196	71.42263	1.127449	177.1968	81.32875	0.459959
DH_1_193	5.846211	71.85408	73.20724	1.642394	182.6647	82.18446	0.458677
DH_1_194	5.223488	73.79094	74.23662	1.008612	181.5711	84.50688	0.460471
DH_1_195	6.152278	73.50539	74.88879	1.568078	179.8617	85.34953	0.462617
DH_1_196	5.242204	71.37292	72.69104	1.556243	174.2911	77.56392	0.456653
DH_1_197	5.369617	71.38502	73.46706	2.013363	177.0224	80.59212	0.458569
DH_1_199	6.871118	72.10027	72.34783	0.972997	184.5563	86.16772	0.461074
DH_1_2	6.164452	74.09137	74.95448	1.333383	180.5142	82.19879	0.45976
DH_1_201	5.103938	73.13354	73.78958	1.049729	170.9318	81.63382	0.461658
DH_1_202	5.590767	71.75535	73.80798	1.92752	177.3182	81.87979	0.461139
DH_1_203	5.947536	70.44254	71.28716	1.314229	187.9833	84.4389	0.459882
DH_1_204	5.649227	74.11363	76.16267	1.93263	180.83	86.75624	0.46339
DH_1_205	6.580768	73.23242	74.67367	1.569871	173.7097	80.66317	0.461144

Taxa	GY	AD	SD	ASI	PH	EH	EPO
DH_1_206	5.035796	72.15849	74.3342	2.050907	171.7619	80.59463	0.460724
DH_1_207	6.308615	72.41657	74.24677	1.835505	181.8819	88.72508	0.464999
DH_1_208	5.160324	74.42075	76.02804	1.803453	181.795	84.28909	0.461645
DH_1_209	4.80405	73.03913	73.86694	1.255353	169.4944	80.78936	0.461073
DH_1_210	6.065506	72.0699	73.16823	1.307368	180.2755	82.34217	0.459772
DH_1_211	6.003633	72.87299	73.53583	1.042314	184.8436	86.44955	0.46138
DH_1_212	5.678666	72.25626	73.73639	1.616369	170.7436	81.26134	0.462296
DH_1_215	5.851006	71.60683	73.99344	3.668717	174.9727	80.22069	0.459446
DH_1_216	5.719504	72.59832	74.45564	1.812714	182.6791	81.03942	0.457716
DH_1_217	5.89248	72.25653	74.29605	2.013899	176.9446	82.5365	0.460107
DH_1_218	6.343828	71.88118	73.20743	1.597203	177.5006	82.02554	0.459814
DH_1_219	5.812884	73.11617	74.41306	1.505432	171.8266	80.42492	0.460578
DH_1_22	5.681785	72.63928	74.54359	1.899154	180.7973	80.94051	0.458659
DH_1_23	5.452847	71.92038	74.03286	2.032772	172.8967	79.06194	0.458964
DH_1_24	6.676374	73.79186	75.53604	1.793527	183.1516	83.28917	0.460043
DH_1_25	5.753023	72.66548	74.28701	1.666073	168.8776	80.92838	0.462455
DH_1_26	5.258858	72.14662	73.22815	1.294041	177.6963	80.99939	0.459303
DH_1_27	5.05809	74.53559	76.17718	1.774909	170.9537	78.87287	0.459302
DH_1_28	6.193575	69.15924	70.70476	1.626131	179.0339	81.17216	0.458806
DH_1_29	5.344052	70.34786	71.12313	1.214281	170.4364	80.1461	0.460571
DH_1_3	5.456016	71.63657	74.05016	2.277354	173.8756	77.44385	0.456655
DH_1_32	5.395452	73.37073	74.8403	1.588604	174.898	76.30931	0.454937
DH_1_33	6.001301	70.95661	73.04086	1.998434	176.4947	82.36226	0.461177
DH_1_35	5.538635	71.63395	73.44498	1.651561	170.7626	77.82925	0.458047
DH_1_37	5.957147	72.10978	72.64971	1.01906	178.2406	82.521	0.460433
DH_1_38	4.688291	72.99404	74.29811	1.518332	177.3754	79.73494	0.458453
DH_1_39	4.5962	73.39316	75.26089	1.898281	175.8223	81.456	0.459956
DH_1_4	5.496354	71.90861	73.0438	1.387027	181.0472	83.66868	0.461005
DH_1_41	5.740726	74.12822	75.06645	1.258389	184.0409	82.30893	0.458517
DH_1_42	5.331539	71.87791	73.50483	1.653508	176.205	82.97759	0.461041
DH_1_44	6.053572	72.88176	73.79756	1.21703	181.2659	83.1278	0.460241
DH_1_45	5.711616	72.38098	73.62929	1.47451	181.0403	80.89912	0.458176
DH_1_46	5.53191	73.6166	74.80241	1.398391	177.4474	80.50996	0.458505
DH_1_47	6.046975	72.75173	73.52992	1.142531	185.7113	85.76246	0.461176

Taxa	GY	AD	SD	ASI	PH	EH	EPO
DH_1_49	6.128616	72.09728	73.41751	1.550364	184.8704	85.13072	0.460714
DH_1_5	5.784317	71.0742	72.49713	1.615766	168.1264	73.28798	0.454438
DH_1_50	5.85034	71.97815	72.95732	1.261467	181.9083	84.71264	0.462505
DH_1_51	6.159716	72.64652	74.7332	2.036606	176.3127	81.30641	0.459765
DH_1_52	6.027405	72.35376	74.09694	1.866956	177.9121	83.87537	0.461541
DH_1_53	6.827987	71.33737	72.52751	1.481299	184.9606	86.87128	0.461844
DH_1_55	5.492139	72.91608	73.94981	1.290221	175.8355	79.75315	0.458809
DH_1_57	6.568949	71.27602	72.45779	1.428581	179.2635	82.06267	0.459627
DH_1_58	5.829828	70.50969	72.01606	1.597015	166.1057	79.4492	0.462881
DH_1_59	6.022077	73.54963	74.81073	1.413117	186.0544	87.314	0.462171
DH_1_6	5.160444	72.92817	74.75959	1.952802	178.199	78.74338	0.456948
DH_1_60	5.43566	71.25174	73.03494	1.867632	172.8026	83.2617	0.462796
DH_1_61	6.179921	71.58239	73.52732	1.899556	177.3175	84.54177	0.46174
DH_1_63	6.324962	72.00078	73.47986	1.588003	175.583	81.25542	0.459948
DH_1_65	5.480876	74.79219	75.62964	1.17891	170.6681	80.75381	0.461181
DH_1_66	5.626357	72.41154	73.63347	1.429343	180.2327	80.90108	0.458069
DH_1_67	6.157217	71.587	73.75923	2.046461	179.9143	86.84431	0.464028
DH_1_68	4.990007	73.02632	74.525	1.50825	177.5223	78.85111	0.457694
DH_1_69	5.929957	73.28966	74.2911	1.227278	173.62	75.8513	0.456122
DH_1_7	5.557735	71.29187	72.62716	1.497423	181.5433	80.53589	0.457892
DH_1_70	6.234883	72.03686	72.69685	1.13984	179.9935	82.34678	0.458781
DH_1_72	5.357224	73.01233	74.25412	1.43061	179.772	79.0707	0.456477
DH_1_73	6.169901	70.89791	72.56036	1.75504	171.5262	76.62619	0.457667
DH_1_74	5.128636	72.99733	74.6444	1.87578	175.0292	80.15524	0.459905
DH_1_77	4.99589	74.03703	75.31268	1.515631	177.0751	79.09434	0.457993
DH_1_78	6.689564	71.55999	73.13277	1.737763	185.7332	83.78605	0.459572
DH_1_79	5.826117	70.95848	72.63597	1.751427	174.6378	78.65358	0.457137
DH_1_8	6.02286	70.44433	71.87088	1.558275	170.9491	76.15082	0.458001
DH_1_80	5.843613	70.50175	72.19334	1.76699	172.1926	79.2132	0.459822
DH_1_81	5.862094	73.22103	74.08793	1.233251	180.0721	86.25003	0.462354
DH_1_82	5.973844	72.90362	74.23816	1.595872	183.4069	84.78235	0.460719
DH_1_83	5.910262	71.44082	72.17869	1.058372	181.283	86.16867	0.462924
DH_1_84	5.220748	70.66241	72.03578	1.515714	175.8037	80.14324	0.458369
DH_1_85	5.508044	71.92454	73.16267	1.508728	176.1162	80.89797	0.459333

Taxa	GY	AD	SD	ASI	PH	EH	EPO
DH_1_86	5.944893	71.58404	73.59746	2.005205	184.2656	86.68386	0.46213
DH_1_87	5.848364	72.06473	74.24748	2.037529	176.329	74.90838	0.454542
DH_1_88	5.543848	71.83424	72.7327	1.183261	174.3714	80.20416	0.459609
DH_1_9	5.54011	70.05089	71.31465	1.604397	177.4582	79.07053	0.458124
DH_1_90	5.497296	73.51822	74.73545	1.399657	182.6311	84.97876	0.460751
DH_1_91	6.101034	70.65234	72.26238	1.750082	173.6958	81.1698	0.460563
DH_1_92	6.616206	71.38506	72.75712	1.530235	190.1927	90.44623	0.462977
DH_1_93	6.011899	71.65795	73.16836	1.633637	177.4262	82.70885	0.461026
DH_1_94	5.708654	72.20726	73.47012	1.410446	176.4362	83.62101	0.461205
DH_1_95	5.519966	73.42312	74.43655	1.313979	179.9846	85.13221	0.462141
DH_1_96	5.131503	73.88934	75.03712	1.402587	174.0455	80.39798	0.460058
DH_1_97	5.6268	71.32657	72.68544	1.482078	175.7386	81.55183	0.460262
DH_1_98	5.145993	74.33809	75.86204	1.652901	181.8243	84.10048	0.460258
DH_1_99	5.773123	73.07114	74.09317	1.285064	190.2701	86.21211	0.459504
DH_10_1	5.296543	70.74144	71.75222	1.28145	172.5437	81.84597	0.462133
DH_10_10	5.897475	72.88482	73.45097	1.054597	171.0649	82.74029	0.462816
DH_10_103	5.411455	69.94286	71.56098	1.676734	173.3388	79.85278	0.460638
DH_10_104	5.606308	67.29744	68.79123	1.428974	174.79	85.14457	0.46439
DH_10_12	5.823521	68.85755	69.68628	1.059803	171.0684	77.70149	0.458783
DH_10_13	5.568713	72.65957	74.02593	1.52199	173.8531	81.37333	0.46102
DH_10_19	5.641662	69.27607	70.41395	1.417159	162.8238	74.28548	0.457125
DH_10_2	4.382823	69.78856	71.49689	1.753101	172.694	81.06636	0.46134
DH_10_26	4.961273	72.57334	74.47974	1.845872	176.0605	78.88923	0.457124
DH_10_41	5.221423	71.59735	73.07694	1.563004	176.0198	78.30606	0.457836
DH_10_42	5.295678	69.35307	70.88903	1.469874	161.7864	76.00627	0.460823
DH_10_43	5.119142	67.99606	69.57137	1.491299	167.2682	81.14345	0.462793
DH_10_44	5.099752	71.66806	72.82374	1.406234	171.4308	80.56462	0.461215
DH_10_46	5.443277	69.34985	70.8688	1.647439	181.439	84.2216	0.460582
DH_10_49	4.840334	69.49672	71.78176	2.038796	171.3719	79.52674	0.460247
DH_10_50	5.574017	70.11034	71.18644	1.301483	174.6802	79.02365	0.457922
DH_10_52	4.938405	70.23024	71.36365	1.360679	167.5815	78.7598	0.459793
DH_10_53	4.746171	72.25948	73.92043	1.758381	174.9514	80.1506	0.458998
DH_10_54	5.533193	68.94842	69.88126	1.254808	174.9685	81.07043	0.460095
DH_10_62	4.89126	69.26206	71.04211	1.771842	175.9336	80.3463	0.459588

Taxa	GY	AD	SD	ASI	PH	EH	EPO
DH_10_64	4.505108	71.86602	73.63203	1.792265	173.9118	86.18655	0.464833
DH_10_65	4.938089	69.14012	71.55159	2.180316	170.5158	81.18614	0.461254
DH_10_67	4.385694	71.47559	73.20875	3.22519	170.2188	80.21814	0.460829
DH_10_68	5.063754	71.37108	72.51953	1.308267	177.9999	84.55063	0.46259
DH_10_7	5.403278	68.11813	68.51338	0.817727	167.9911	80.53354	0.461687
DH_10_74	5.21556	71.85002	73.45031	1.626344	174.8166	84.534	0.463383
DH_10_78	4.983611	69.97487	71.57964	1.662577	167.0223	73.11652	0.455045
DH_10_84	5.422155	70.39098	70.92146	0.973554	178.5991	87.39073	0.464671
DH_10_86	5.103691	69.00569	70.7339	1.756585	176.8942	80.48775	0.459428
DH_10_87	5.492869	71.19238	71.39602	0.646396	171.8777	78.616	0.458387
DH_10_9	4.641702	70.02459	71.82905	1.713526	175.1122	82.53572	0.462254
DH_10_91	5.95533	67.89822	69.25289	1.491838	177.044	80.11864	0.458245
DH_10_93	5.3414	70.65075	71.76857	1.378337	183.1504	82.40918	0.458812
DH_10_97	6.020952	68.72286	69.5182	1.042264	166.1861	76.9444	0.459888
DH_10_98	5.320075	69.56966	71.40425	3.248896	173.572	82.90661	0.463378
DH_2_1	5.252693	70.87121	72.05162	1.511463	170.5356	82.69259	0.46279
DH_2_102	5.004433	71.47765	72.5311	1.44933	172.5859	76.60025	0.457059
DH_2_11	5.149083	71.61217	72.86695	1.55237	174.6928	80.92814	0.460125
DH_2_14	4.793529	70.44419	72.17957	1.799904	168.7307	74.49145	0.455648
DH_2_15	5.094167	70.90673	73.50123	2.354941	173.2601	79.37237	0.458616
DH_2_17	5.275718	71.0182	72.97176	1.872435	178.5769	80.83723	0.459351
DH_2_18	5.485764	70.20079	72.38135	2.109416	172.8789	78.85845	0.458299
DH_2_2	5.333815	71.39187	77.15402	4.500214	183.543	85.00474	0.461203
DH_2_21	5.465621	69.49028	72.67884	2.741719	172.306	80.17657	0.460598
DH_2_24	5.15013	71.71918	73.93015	2.070466	173.028	79.42908	0.459485
DH_2_25	4.27368	71.83042	74.39982	2.289793	175.361	79.4051	0.459552
DH_2_27	5.190005	71.96017	73.25108	1.49965	177.3554	83.11961	0.460985
DH_2_28	5.239981	71.20644	73.00711	1.822961	165.095	76.97524	0.460029
DH_2_3	5.019458	71.10376	73.18821	2.182431	172.3329	78.55142	0.459405
DH_2_31	4.977806	70.97341	72.37877	1.554776	169.5059	77.5009	0.45829
DH_2_33	5.560678	71.17152	73.76165	2.293291	174.6883	80.86533	0.460555
DH_2_34	5.329445	71.39326	73.69538	2.140959	174.7892	78.30195	0.459073
DH_2_35	5.084504	72.06966	74.7145	2.388524	173.2553	78.14363	0.456637
DH_2_36	4.917349	71.24165	74.25886	2.577116	174.0063	75.21568	0.454591

Taxa	GY	AD	SD	ASI	PH	EH	EPO
DH_2_37	4.771978	72.57038	75.24337	2.311851	169.5757	74.2011	0.457719
DH_2_38	5.529313	71.44078	75.69326	3.504203	181.3792	85.14137	0.461905
DH_2_39	5.151804	72.03346	75.71139	3.11973	172.5295	77.69785	0.458928
DH_2_42	5.145788	71.7085	73.60825	1.900244	173.5012	84.47666	0.463567
DH_2_43	5.300403	73.29909	74.6429	1.468593	171.3161	80.90265	0.460537
DH_2_48	4.954087	72.65218	76.57344	3.270151	172.9769	80.09915	0.459625
DH_2_49	5.38234	70.64768	72.9882	2.094565	181.4648	80.12003	0.457743
DH_2_5	5.536285	70.2439	72.3083	1.94023	177.8103	84.04864	0.461631
DH_2_50	5.141321	69.62019	72.83921	2.68735	174.8064	79.71812	0.457221
DH_2_53	5.229771	72.7386	74.71793	2.117067	175.2674	78.47506	0.458314
DH_2_58	4.650884	71.29916	73.83355	2.279748	176.336	79.39917	0.458889
DH_2_59	4.888849	72.5012	74.62393	2.029633	173.9992	83.21139	0.46206
DH_2_6	4.866577	71.43721	74.0062	2.397102	174.5706	78.71155	0.457862
DH_2_61	5.302303	70.80216	72.40939	1.797711	169.6811	77.67276	0.459238
DH_2_62	5.245932	71.18044	73.54581	2.235685	170.8911	76.08378	0.456936
DH_2_65	5.055952	71.39642	74.76184	2.900149	175.7644	81.74813	0.460511
DH_2_66	4.902166	71.62963	73.34156	1.847277	163.6906	77.71649	0.461348
DH_2_67	6.253443	70.9709	72.3941	2.367057	181.4016	84.56688	0.460722
DH_2_70	5.204138	68.79364	71.58419	2.516508	175.3559	83.61648	0.462767
DH_2_71	4.257032	73.67735	75.95568	2.067985	171.211	76.5991	0.457973
DH_2_72	5.950169	70.69345	72.50843	1.913075	170.9116	80.21319	0.46083
DH_2_73	5.471074	71.48441	73.45012	1.921171	171.8622	80.45117	0.461157
DH_2_74	4.848306	72.94004	75.17134	2.067592	169.6353	76.09047	0.45662
DH_2_75	5.202593	71.37086	72.94628	3.860702	169.3404	80.10776	0.462204
DH_2_78	5.184986	70.90004	73.0895	2.087397	180.8875	80.85128	0.458945
DH_2_80	5.337302	71.3175	73.59482	2.155055	171.5887	78.65597	0.458946
DH_2_81	5.006032	72.58518	76.35733	3.104322	173.4968	77.34735	0.458724
DH_2_84	5.607937	70.31274	72.97626	2.29642	173.6496	79.38792	0.45971
DH_2_85	5.132997	72.92093	74.87651	1.924511	178.1756	81.15455	0.459401
DH_2_87	5.426104	71.26739	73.68191	2.246118	171.4664	80.47331	0.461416
DH_2_88	4.811282	72.69128	74.25517	1.666756	175.5161	82.2758	0.460683
DH_2_9	4.985664	72.53811	74.33251	1.702296	176.3913	79.82047	0.458857
DH_2_90	5.383181	71.07274	73.26673	2.113031	175.4311	83.04358	0.461589
DH_2_93	6.183948	69.77111	70.86095	1.318732	170.9285	76.79814	0.457982

Taxa	GY	AD	SD	ASI	PH	EH	EPO
DH_2_96	5.032442	69.08599	72.14414	2.633507	167.2844	73.79585	0.455905
DH_2_97	6.016654	71.13557	72.98038	1.846348	170.3157	76.51332	0.45753
DH_2_98	5.290804	71.72137	74.07205	2.316955	175.1747	78.84876	0.45714
DH_2_99	4.781189	70.27405	72.83862	2.438934	174.5302	79.48514	0.45858
DH_3_1	4.482128	70.95071	72.25218	1.521179	171.5218	78.77104	0.457423
DH_3_10	5.369655	69.91419	71.41444	1.716124	173.4898	80.15036	0.461108
DH_3_101	5.400025	72.23699	74.54911	2.042103	174.4046	79.20764	0.459898
DH_3_102	5.989651	70.53256	71.81952	1.486497	178.1786	82.76576	0.460896
DH_3_103	5.421897	69.44335	71.32678	1.818379	174.4044	79.00151	0.458422
DH_3_105	5.343577	71.07337	73.02869	1.905985	173.559	79.34299	0.459425
DH_3_106	5.698935	71.13207	72.61196	1.640377	175.6481	80.38586	0.459041
DH_3_107	5.671414	70.19307	71.43448	1.572577	175.3342	78.99478	0.457731
DH_3_108	5.657946	71.532	73.22263	1.682618	172.207	78.93623	0.457951
DH_3_109	5.448025	71.12695	72.49596	1.528926	172.5835	77.99533	0.457177
DH_3_11	5.440443	71.19744	72.15111	1.252159	179.6128	78.85153	0.4576
DH_3_110	5.184211	70.67698	73.01782	2.246642	171.102	79.73465	0.459231
DH_3_111	5.101301	71.96813	73.35071	1.559424	173.191	81.29851	0.461696
DH_3_116	5.271457	70.93851	72.89689	1.910393	176.7416	79.95118	0.458023
DH_3_123	4.796655	71.07045	73.0095	1.917377	165.3023	76.97899	0.46011
DH_3_124	5.528789	69.18158	70.54117	1.509375	176.966	81.76797	0.459919
DH_3_125	5.690212	70.23942	71.58951	1.519454	173.3968	82.57965	0.461674
DH_3_126	5.437734	71.67293	73.74963	2.106694	170.0962	78.31481	0.46037
DH_3_129	5.583324	68.8803	70.69349	1.787148	168.9209	79.13051	0.460379
DH_3_13	5.637888	69.74873	72.17171	2.166648	177.752	80.9074	0.458999
DH_3_131	5.385861	69.84833	72.57552	2.338763	168.9429	77.22091	0.459846
DH_3_135	5.465857	70.36356	71.35332	1.360341	181.0292	84.34997	0.461329
DH_3_136	5.545979	69.69322	71.35784	1.692063	174.6565	81.61738	0.460948
DH_3_137	5.745648	71.10189	72.89695	1.773461	174.5458	79.5845	0.459061
DH_3_138	5.263327	73.04073	75.54205	2.37558	172.2435	74.26282	0.45423
DH_3_139	5.457687	69.98816	71.90166	1.763818	175.0528	81.89686	0.461847
DH_3_14	6.062886	71.14941	74.45342	2.881433	178.8422	82.21984	0.459973
DH_3_142	5.058309	69.72335	71.62743	1.897751	165.0665	73.93666	0.456105
DH_3_143	5.285155	70.96724	72.39561	1.556491	176.6464	78.29143	0.458186
DH_3_145	5.598639	71.01646	73.0629	1.939906	173.648	82.48382	0.46245

Taxa	GY	AD	SD	ASI	PH	EH	EPO
DH_3_147	5.411019	69.706	71.68244	1.871874	172.5722	79.43206	0.458852
DH_3_15	5.397052	70.4363	72.55867	2.095514	172.7335	79.94685	0.460601
DH_3_151	5.957444	68.85276	70.04351	1.46628	175.7937	79.29524	0.457846
DH_3_16	5.074551	71.33603	73.58524	1.808508	172.7198	75.23991	0.456112
DH_3_18	5.693134	71.40156	72.62849	1.391886	168.8993	81.38861	0.461637
DH_3_19	5.397719	70.21222	72.47738	2.139141	176.5849	77.49701	0.457316
DH_3_2	6.010761	71.1722	72.92281	1.781115	175.6036	83.76566	0.462986
DH_3_20	4.984844	69.7474	71.95369	2.026658	173.6668	81.10658	0.459798
DH_3_22	5.565609	70.30784	71.86319	1.64332	178.3341	80.28883	0.45886
DH_3_23	5.392139	69.48297	70.61452	1.163774	171.4816	79.07407	0.460155
DH_3_26	5.674405	70.1301	71.39575	1.48769	166.008	76.45339	0.45939
DH_3_27	5.810118	67.83253	69.67831	1.783317	172.4305	80.49064	0.46107
DH_3_29	5.59882	69.22587	69.90191	1.075642	167.7146	78.2764	0.460167
DH_3_3	5.620131	69.94384	71.98944	1.954973	176.8503	83.80911	0.461493
DH_3_30	5.754437	69.43058	72.05646	2.304087	170.4957	77.31652	0.457933
DH_3_31	5.21877	69.81954	70.90811	1.358471	173.4097	80.32208	0.460855
DH_3_32	4.883664	71.24695	73.47076	2.064454	173.0542	81.46805	0.460623
DH_3_34	5.023803	69.6276	72.07595	2.18242	164.7406	77.48393	0.460102
DH_3_36	5.811552	70.283	71.58183	1.445376	181.6908	80.40172	0.45885
DH_3_38	5.791584	70.57511	72.16446	1.53766	177.7831	83.03486	0.462018
DH_3_39	5.37291	70.33102	72.32074	1.819581	175.3271	77.65711	0.457335
DH_3_4	5.70542	71.38575	73.24309	1.815959	178.463	83.07511	0.461022
DH_3_41	5.608678	70.60453	72.32322	1.686888	170.8383	79.52184	0.461073
DH_3_43	5.110194	70.92984	71.72337	1.223158	168.1873	74.9683	0.456709
DH_3_46	5.321399	70.75451	72.7601	1.953244	173.4991	77.21022	0.457004
DH_3_47	4.971349	70.69146	72.09731	1.487205	172.8588	76.7095	0.454781
DH_3_48	5.737967	68.05989	69.17344	1.274959	172.733	78.12419	0.456349
DH_3_5	5.921466	69.55922	72.19156	2.319659	173.7281	80.65522	0.461167
DH_3_50	5.455316	70.72767	72.90744	1.99183	178.5713	82.40656	0.459579
DH_3_53	5.83705	69.70913	70.93384	1.340326	175.449	82.34458	0.461106
DH_3_56	5.407135	69.02371	70.51902	1.636044	176.4417	82.60478	0.460932
DH_3_57	5.59089	70.95798	72.26809	1.409433	181.0959	81.72873	0.458355
DH_3_58	5.473437	70.46011	72.26718	1.7849	177.5127	81.19359	0.459058
DH_3_59	5.410075	71.2777	72.60748	1.551825	178.8241	81.94912	0.45984

Taxa	GY	AD	SD	ASI	PH	EH	EPO
DH_3_6	5.745141	69.36857	70.78214	1.457173	180.0234	82.83011	0.460276
DH_3_60	5.355509	70.5746	72.42547	1.881655	167.3643	74.96323	0.456065
DH_3_61	5.234457	70.81286	73.05167	2.041796	161.4618	70.93467	0.453804
DH_3_62	5.614893	72.10763	73.9168	1.803327	175.8576	79.75593	0.459277
DH_3_63	5.617377	69.66785	71.54835	1.823384	169.5192	76.59197	0.457816
DH_3_64	5.19084	70.45172	72.15617	1.685042	174.26	80.23221	0.459438
DH_3_65	5.811607	70.3243	72.53984	2.061072	176.0129	81.00956	0.460301
DH_3_66	5.663357	70.72316	72.51822	1.797243	169.5397	75.24536	0.456687
DH_3_67	5.013534	69.61154	70.75798	1.322715	171.8483	75.2273	0.455148
DH_3_68	5.205594	70.89796	72.79598	1.953504	168.1157	75.68444	0.457465
DH_3_69	5.593244	69.46634	70.46845	1.260082	178.5559	82.9761	0.460555
DH_3_7	5.474674	70.40191	72.08663	1.827134	174.8957	77.92066	0.457292
DH_3_72	5.597492	73.0663	76.32957	2.85701	176.5327	80.62283	0.459678
DH_3_74	5.820388	71.26526	73.13593	1.716831	179.4181	81.15242	0.458374
DH_3_75	5.056176	69.26429	70.44053	1.457442	178.395	80.68739	0.458627
DH_3_77	5.537478	69.82782	72.02005	2.02352	171.4578	77.26838	0.457979
DH_3_80	5.80093	70.15039	71.44918	1.494717	174.6979	80.69772	0.460159
DH_3_81	4.655754	70.46434	72.708	2.060167	168.4475	75.78232	0.457383
DH_3_82	5.100988	69.48765	71.15599	1.790835	174.4516	82.5227	0.461685
DH_3_83	5.316088	72.38271	73.47639	1.471654	176.3371	76.75227	0.454477
DH_3_84	5.403784	69.36706	71.38884	2.029733	159.7189	80.6146	0.520652
DH_3_85	5.219183	70.31895	70.28265	0.622247	181.5071	84.55377	0.462409
DH_3_86	5.411371	70.72854	72.30627	1.686259	168.0584	75.60604	0.457294
DH_3_87	5.3067	68.55169	69.96641	1.534748	176.1076	83.91572	0.463242
DH_3_88	5.723821	70.94119	72.26849	1.448476	175.6465	79.72324	0.458313
DH_3_89	4.332836	69.99486	71.945	1.913868	158.931	73.3422	0.45827
DH_3_9	4.900936	73.62891	75.40629	1.703869	171.3422	79.58421	0.458873
DH_3_91	5.632143	70.15444	71.39207	1.498769	173.8118	78.63582	0.458527
DH_3_94	5.941257	71.09297	73.68598	2.293768	181.0191	85.82099	0.462857
DH_3_95	5.313892	70.81793	72.38438	1.662293	169.2964	82.04923	0.462163
DH_3_96	6.464447	69.8671	71.48335	1.736311	181.1389	87.3906	0.464242
DH_3_98	5.253353	71.26293	73.47113	2.025854	175.8837	77.93753	0.458182
DH_3_99	5.865482	71.6646	72.73603	1.280087	186.491	87.87485	0.463398
DH_4_11	5.136723	72.55976	75.51863	2.549053	174.0406	76.92905	0.457064

Taxa	GY	AD	SD	ASI	PH	EH	EPO
DH_4_12	6.434705	70.38703	71.70639	1.438863	182.9799	80.45916	0.457164
DH_4_13	5.458439	69.17212	71.11767	1.907293	174.0216	80.76216	0.460959
DH_4_14	6.142314	69.57024	71.00204	1.533185	175.3018	83.67678	0.4618
DH_4_19	5.549151	71.83178	73.21182	1.432852	177.2489	80.00543	0.457912
DH_4_20	5.603352	71.44321	72.64336	1.371722	176.239	80.60653	0.459236
DH_4_22	5.478959	67.59402	69.01252	1.538778	172.0189	83.20565	0.463019
DH_4_23	4.330946	70.98432	73.77229	2.441846	164.8401	74.97575	0.455973
DH_4_24	4.840112	69.37974	70.98571	1.579868	173.9118	79.9303	0.460006
DH_4_25	5.821051	72.26915	73.81085	1.674536	174.4155	79.89806	0.45864
DH_4_26	5.714397	70.26351	72.62876	2.023644	173.5153	76.68042	0.457377
DH_4_27	5.514417	71.44841	73.15609	1.616644	177.106	76.45684	0.455588
DH_4_28	6.141555	70.30015	72.1301	1.862156	180.0089	81.17417	0.458411
DH_4_29	5.733752	70.32018	71.52725	1.568299	168.303	77.7308	0.459205
DH_4_3	4.949373	70.66925	72.4692	1.840918	171.8786	78.6849	0.458878
DH_4_30	4.887545	70.54263	72.07384	1.66242	172.5278	80.91279	0.460237
DH_4_31	5.762024	71.49122	73.25249	1.803256	184.293	84.76384	0.460638
DH_4_32	5.634846	70.84857	72.93079	1.948133	176.8369	83.0065	0.460942
DH_4_33	6.105106	71.16798	72.77985	1.657125	176.1579	82.52421	0.461681
DH_4_34	5.330448	70.763	73.34945	2.355636	183.9349	82.62507	0.457972
DH_4_35	5.143686	71.96361	74.4466	2.284141	182.6658	85.72852	0.46089
DH_4_36	5.199654	70.58247	72.63368	1.946183	181.5908	83.58014	0.46013
DH_4_37	5.604657	71.57722	73.63988	1.941942	177.2865	80.27023	0.458839
DH_4_38	5.42817	70.40146	71.81227	1.610555	170.0823	79.23445	0.461552
DH_4_39	5.157081	70.85661	74.37652	2.958053	178.3187	80.92197	0.458689
DH_4_4	5.214225	69.84891	72.04034	2.17342	164.5457	72.97039	0.454995
DH_4_42	5.418179	72.82722	74.53713	1.777263	183.9252	83.66709	0.45861
DH_4_44	5.719321	71.04946	72.62609	1.560074	183.2127	81.41602	0.457729
DH_4_45	5.540945	69.66041	71.45291	1.862727	180.3076	80.95875	0.457217
DH_4_46	5.439401	72.21257	74.06366	1.877189	183.3008	83.89899	0.459275
DH_4_47	5.85785	69.94817	71.98503	1.917951	180.3241	82.28814	0.45932
DH_4_49	5.590906	71.73092	72.99551	1.51384	172.6902	79.77251	0.459724
DH_4_53	5.228331	70.05128	71.59778	1.703796	170.0765	79.00477	0.459648
DH_4_54	5.543357	71.38112	72.95599	1.643561	176.8749	82.68214	0.461238
DH_4_8	5.81813	70.13986	71.87639	1.878189	170.7453	78.84657	0.459719

Taxa	GY	AD	SD	ASI	PH	EH	EPO
DH_4_9	5.366185	71.46723	73.72973	2.149304	184.6811	80.25718	0.456913
DH_5_1	4.957859	71.98871	73.43949	1.486839	176.5173	84.01177	0.461851
DH_5_10	3.865948	76.60018	78.15356	4.015617	175.076	80.82616	0.459635
DH_5_11	4.964819	74.26116	76.63181	2.11667	177.2086	83.06716	0.460575
DH_5_12	5.363807	69.83694	71.01992	1.387617	180.1286	81.14127	0.459284
DH_5_13	5.819868	71.66364	72.83819	1.349081	184.0247	85.51822	0.460353
DH_5_15	6.296634	73.18558	74.72002	1.568987	180.8467	84.68236	0.462558
DH_5_17	5.448812	73.15052	74.12201	1.427924	177.5629	85.59295	0.462486
DH_5_2	4.786634	72.30393	73.42318	1.389203	170.7746	78.2467	0.460097
DH_5_21	5.401703	76.37113	79.19853	4.368952	167.2973	76.12845	0.45693
DH_5_22	3.733741	76.12899	77.89992	3.973025	171.6711	77.85177	0.457747
DH_5_23	5.792837	69.2108	71.09981	1.881319	176.7525	81.38833	0.461162
DH_5_26	4.570347	72.41083	75.44441	2.565496	177.6477	84.05524	0.461594
DH_5_27	5.568973	71.78321	73.56722	1.803549	177.6344	80.59088	0.460265
DH_5_28	3.149006	76.199	76.67129	2.534128	162.9611	76.80863	0.458899
DH_5_3	5.255951	72.44629	74.09753	1.684812	179.8292	82.34598	0.460976
DH_5_33	5.570084	72.15999	74.01332	1.870831	176.8985	78.96033	0.457556
DH_5_35	5.284484	73.77363	74.95214	1.368462	177.3555	82.84042	0.461454
DH_5_36	5.031439	72.1474	73.11861	1.373531	168.5688	78.7058	0.459691
DH_5_37	5.385232	70.23877	72.58092	2.15687	171.8114	78.87394	0.458906
DH_5_38	5.367502	71.48845	72.61795	1.316352	164.7792	79.94774	0.464267
DH_5_4	5.593928	74.21808	75.57916	1.540405	179.2006	81.52168	0.458891
DH_5_40	5.845159	72.32919	73.53004	1.474669	185.0848	88.80205	0.46399
DH_5_41	6.10121	71.69566	73.67946	1.998198	184.4098	91.84021	0.4667
DH_5_43	4.269114	77.42018	77.71041	4.852905	168.9568	74.30535	0.458659
DH_5_44	5.896391	71.7151	73.70272	1.920663	183.3552	81.2179	0.457512
DH_5_45	5.156577	73.29979	74.779	3.877548	172.8386	81.53628	0.459685
DH_5_47	5.835211	71.6804	72.93129	1.467014	176.0721	79.72156	0.457271
DH_5_48	5.402852	71.51752	73.17922	1.611996	168.2116	74.60529	0.456006
DH_5_5	4.642073	71.51696	73.37985	1.87416	174.8855	80.20537	0.459358
DH_5_51	5.372846	72.13035	73.08123	1.342018	182.401	84.09219	0.459502
DH_5_53	6.062385	70.54577	72.10733	1.567447	180.3171	84.44245	0.460922
DH_5_56	4.901479	73.10987	74.16392	1.36262	173.1795	81.09899	0.460089
DH_5_57	6.271845	71.4387	72.01995	0.931927	178.7021	85.36543	0.462461

Taxa	GY	AD	SD	ASI	PH	EH	EPO
DH_5_58	5.160682	71.77656	73.64945	1.868176	181.9803	85.5336	0.463495
DH_5_59	5.66996	72.12298	74.09505	1.92159	177.5641	79.07885	0.457824
DH_5_60	3.442569	76.25391	77.83711	3.889734	169.5759	79.74792	0.461268
DH_5_62	5.976878	69.84007	71.32104	1.639851	180.1548	83.06116	0.458915
DH_5_63	5.113787	73.39638	75.27881	1.810392	176.4437	80.0729	0.458186
DH_5_65	4.012736	72.15305	73.37208	1.430471	173.4814	76.6015	0.45621
DH_5_67	5.535746	73.6717	74.99885	1.430725	173.303	82.4801	0.461179
DH_5_69	4.043553	78.80763	78.69235	4.491386	156.6806	74.93286	0.458087
DH_5_70	5.650912	73.35366	74.45789	1.395581	182.3363	89.56009	0.46549
DH_5_73	5.213049	72.39149	74.00554	1.671447	176.7722	78.79422	0.458914
DH_5_74	5.528844	74.11906	75.32602	1.498803	171.9457	83.87838	0.464894
DH_5_75	3.844019	73.77239	80.24396	5.603524	171.3953	80.55521	0.460343
DH_5_76	5.159405	72.07455	73.96577	1.89555	174.7466	76.76779	0.457943
DH_5_77	5.308407	73.20481	74.99156	1.80358	177.8441	82.064	0.461515
DH_5_8	5.478764	72.16746	73.72722	1.660147	175.2671	81.449	0.459536
DH_5_9	4.13217	76.91915	77.97337	1.821696	166.7874	75.63538	0.45733
DH_6_1	5.192606	70.85212	72.30558	1.526588	169.2797	77.54523	0.45839
DH_6_100	5.550604	69.74926	71.4363	1.686005	173.7461	79.12944	0.45866
DH_6_101	5.631115	69.01497	74.47451	3.827361	173.3922	81.39049	0.460747
DH_6_105	6.234026	68.75435	70.59872	1.862955	181.8247	86.84866	0.462784
DH_6_106	5.344016	69.49676	71.25771	1.837863	173.2324	77.07548	0.456787
DH_6_108	5.258828	68.44156	70.37504	1.915452	176.7492	79.91154	0.458503
DH_6_112	5.340936	69.93147	71.82657	1.890497	170.5907	80.94092	0.461206
DH_6_113	5.490729	70.56535	72.87758	2.151667	176.8446	77.61444	0.456581
DH_6_114	5.559481	68.58179	70.58657	1.857528	177.0246	84.43431	0.462317
DH_6_116	5.376347	70.24598	71.71066	1.665043	171.6081	73.95322	0.455737
DH_6_120	5.614218	68.13352	69.48144	1.459555	167.9422	74.53737	0.455851
DH_6_13	5.508437	67.57348	68.70959	1.275639	170.185	78.16167	0.458839
DH_6_14	5.623675	68.13215	70.59886	2.112393	180.6205	85.70825	0.462238
DH_6_15	5.289205	70.78398	72.40321	1.746505	172.0643	78.45813	0.458062
DH_6_16	5.721635	68.84607	70.26419	1.503186	169.5274	75.63343	0.456923
DH_6_17	5.571437	69.66609	71.40364	1.771652	181.1265	86.12007	0.463047
DH_6_19	5.55729	68.96889	70.6408	1.55898	175.6215	81.15274	0.460556
DH_6_20	5.585086	69.32491	70.96263	1.664217	180.2262	82.0023	0.459598

Taxa	GY	AD	SD	ASI	PH	EH	EPO
DH_6_22	6.090855	69.6962	71.35755	1.674884	178.3776	82.5644	0.460105
DH_6_23	5.29527	70.02483	71.25299	1.497811	178.5958	79.68515	0.457885
DH_6_26	5.467893	67.56396	69.81315	2.039258	171.7264	77.56054	0.458333
DH_6_28	5.978552	68.51409	69.35768	1.08687	174.9844	83.13017	0.462271
DH_6_29	5.009039	69.02381	70.19562	1.355135	166.2782	75.35677	0.456974
DH_6_30	5.10939	68.89099	71.36164	2.133151	174.4368	81.71634	0.460894
DH_6_31	6.411659	68.12801	69.62739	1.536602	172.5222	78.48444	0.45879
DH_6_33	5.439374	70.25553	72.1112	1.876149	168.1433	74.86095	0.456615
DH_6_35	5.113378	68.7651	72.16713	2.729673	172.092	78.47934	0.458809
DH_6_38	4.992346	69.22802	70.80043	1.604388	166.7265	77.17942	0.458954
DH_6_4	5.403708	69.9313	72.54281	2.161803	176.6321	82.1798	0.460563
DH_6_40	5.397316	68.06547	70.0657	1.948558	177.7945	81.94348	0.459789
DH_6_41	5.536131	71.31485	74.14524	2.427603	173.288	83.32682	0.462756
DH_6_43	6.172022	68.50644	70.1325	1.718187	180.7951	82.11517	0.459732
DH_6_45	5.730868	70.60545	72.90112	2.140762	174.715	78.04245	0.457683
DH_6_46	5.057129	71.87845	74.05347	2.071816	166.9113	77.24392	0.458823
DH_6_5	5.639324	68.84959	70.56168	1.666755	174.378	78.67585	0.4583
DH_6_52	5.029526	70.14503	71.64041	1.50906	170.3393	75.39123	0.456616
DH_6_57	5.620584	68.18952	69.63378	1.627399	171.5545	76.80212	0.457479
DH_6_6	5.251038	70.1756	73.39451	2.574204	164.3836	79.74676	0.459427
DH_6_60	5.884051	68.53769	70.0921	1.630983	167.3643	78.93608	0.46058
DH_6_61	4.914296	70.01644	71.59243	1.648806	176.1128	80.84738	0.459019
DH_6_64	6.180288	69.12741	70.52651	1.494607	175.6242	79.31794	0.458469
DH_6_65	6.321553	68.05551	69.13299	1.438741	181.2609	83.73747	0.460854
DH_6_68	5.808616	68.88891	70.66448	1.731615	181.6327	83.07383	0.459509
DH_6_7	5.369639	68.31202	69.78016	1.550366	176.0746	82.03172	0.461166
DH_6_71	6.083299	67.27905	69.75736	2.312151	172.5813	82.26311	0.462282
DH_6_74	5.588935	69.6018	71.41328	1.803895	176.1579	79.77615	0.458367
DH_6_75	4.910143	70.79263	72.55296	1.877511	162.86	74.10564	0.456965
DH_6_76	6.107481	69.74112	71.36736	1.665648	182.7081	78.93592	0.456564
DH_6_79	5.014569	70.04128	71.86156	1.786215	175.856	80.25221	0.459285
DH_6_81	6.501753	70.26229	71.30023	1.378918	175.2019	81.03478	0.460376
DH_6_83	5.501521	68.55734	70.86541	1.923045	165.9589	75.26867	0.457858
DH_6_84	5.613159	68.73241	71.08454	2.10697	168.8634	79.12613	0.45953

Taxa	GY	AD	SD	ASI	PH	EH	EPO
DH_6_85	5.721919	69.90011	71.94805	2.012426	174.9077	81.93178	0.461028
DH_6_86	4.971839	71.2892	73.94829	2.332181	174.5813	80.37981	0.460187
DH_6_90	5.227165	68.44913	70.2808	1.84071	168.8699	80.12054	0.461202
DH_6_91	5.762016	69.10282	70.7153	1.646912	168.3687	76.15915	0.457259
DH_6_93	4.934338	69.34884	71.03716	1.775383	176.4859	78.89725	0.458164
DH_6_94	5.54863	70.96087	73.17764	2.115509	176.2974	79.85722	0.458681
DH_6_95	5.345944	68.88666	69.96288	1.288037	172.1024	82.3404	0.46246
DH_6_97	4.867176	69.23032	70.9629	1.803456	174.8633	76.70338	0.45562
DH_6_98	4.970477	69.72414	71.10956	1.481848	174.3994	79.69917	0.459419
DH_7_10	5.304625	69.98415	72.39522	2.028649	181.5105	85.76623	0.462286
DH_7_102	5.211667	71.70288	73.27695	1.64897	176.3998	83.69971	0.462417
DH_7_12	4.204335	74.47257	76.98564	3.7636	164.7582	78.83347	0.462269
DH_7_14	5.585514	70.46798	72.15153	1.687801	175.77	79.51338	0.459123
DH_7_16	5.241749	69.4869	71.50694	1.94054	179.0874	82.21146	0.460088
DH_7_2	5.602947	71.80282	74.26432	2.175006	175.0246	78.40402	0.458281
DH_7_21	5.835763	70.18467	71.36655	1.394824	178.6441	83.40939	0.46103
DH_7_22	5.357689	71.39171	72.17879	1.212552	186.0163	86.8016	0.461648
DH_7_24	6.048302	69.57763	70.93216	1.363784	181.5712	84.77928	0.461051
DH_7_25	6.254973	70.15366	71.56128	1.523376	181.9075	83.58773	0.460265
DH_7_27	5.488203	71.85903	73.09175	1.439418	176.3624	81.33649	0.460082
DH_7_28	4.946411	70.34695	71.98507	1.651903	175.0516	81.28019	0.45995
DH_7_29	5.729283	70.79459	72.58971	1.795856	175.5206	83.43639	0.462427
DH_7_32	5.322065	69.89188	71.26215	1.5013	181.1338	85.86639	0.462768
DH_7_33	5.836291	72.31283	73.63086	1.427948	172.8821	82.82281	0.462917
DH_7_34	5.737131	73.12048	74.27001	1.471706	178.4871	84.54048	0.462084
DH_7_37	5.435511	72.64123	75.10856	2.273739	181.6544	84.94722	0.461789
DH_7_4	5.689371	71.38448	72.70927	1.458664	173.9266	81.14768	0.460732
DH_7_43	5.575725	73.31879	75.17604	1.904855	180.5813	85.13293	0.462266
DH_7_44	5.924866	71.91404	74.59309	2.414886	181.7061	82.32664	0.459454
DH_7_46	5.628822	72.19514	74.96828	2.508647	182.8558	84.85378	0.461164
DH_7_49	5.397113	70.06684	71.45392	1.530702	181.743	84.66836	0.461023
DH_7_50	5.823132	71.41977	72.86248	1.615	180.0534	83.81775	0.461753
DH_7_51	5.312678	70.88084	71.64441	1.05631	176.0237	80.3724	0.459462
DH_7_52	5.362656	70.30795	71.41757	1.36984	177.3964	82.60413	0.460307

Taxa	GY	AD	SD	ASI	PH	EH	EPO
DH_7_53	5.50214	70.94698	73.23124	2.141512	177.0669	83.2646	0.461366
DH_7_54	4.444783	71.35256	72.03727	1.130904	173.2145	81.51247	0.461044
DH_7_57	4.575146	71.34828	72.84351	1.62752	171.1353	79.35209	0.45977
DH_7_58	5.189508	70.28196	71.74886	1.633236	175.6163	82.52324	0.461216
DH_7_60	5.658902	71.29336	72.14472	1.147645	190.183	85.98493	0.459549
DH_7_62	6.14935	70.43307	71.91597	1.629351	175.304	82.42665	0.461626
DH_7_64	5.789215	69.59658	70.52527	1.270653	183.4618	87.43886	0.462788
DH_7_65	5.570207	69.62426	72.00723	2.072424	171.1734	78.34368	0.459117
DH_7_66	4.832599	71.42946	73.43079	2.004493	174.6865	82.44186	0.460743
DH_7_68	5.136375	72.51012	73.24063	1.051644	183.1939	81.80189	0.45789
DH_7_69	5.815148	70.16259	71.37911	1.315146	173.5571	83.5664	0.462785
DH_7_7	5.650628	71.32773	72.62747	1.584621	172.3213	81.48431	0.462244
DH_7_70	5.580112	70.1039	71.94022	1.8048	178.4467	84.22247	0.461513
DH_7_72	5.405658	70.75026	71.24351	0.90994	177.6102	82.61747	0.460566
DH_7_73	5.540299	71.92999	72.34956	1.115557	180.7682	84.79597	0.461518
DH_7_79	4.898853	71.20314	72.64282	1.55579	174.143	79.47978	0.459288
DH_7_8	5.223338	71.0379	71.97894	1.194217	175.6838	80.03223	0.459569
DH_7_85	5.58098	72.07727	73.62127	1.646821	181.3284	85.97004	0.46214
DH_7_86	5.430812	70.56894	72.34235	1.788619	181.6162	86.71347	0.463233
DH_7_87	5.396361	71.65163	72.75461	1.342274	171.3024	85.51865	0.46578
DH_7_89	6.074201	71.56226	73.06577	1.699261	173.6081	80.32513	0.460222
DH_7_90	4.99859	70.68664	72.24554	1.569729	182.0039	83.82272	0.460561
DH_7_91	5.900366	71.86297	72.91841	1.298778	182.2535	87.75604	0.463567
DH_7_96	5.374727	70.02081	71.4677	1.502329	178.9979	83.59583	0.461318
DH_7_97	5.741455	71.66652	73.31223	1.636389	180.487	86.4284	0.462992
DH_7_98	5.306067	72.96834	73.93097	1.339914	175.2655	79.84158	0.459109
DH_9_1	5.044604	68.50528	70.36445	1.77969	168.201	77.21388	0.458823
DH_9_10	4.449091	70.01811	70.96625	1.25087	177.519	81.6427	0.459931
DH_9_101	5.364843	69.06677	70.80813	1.761623	167.0067	76.85425	0.458228
DH_9_105	4.886115	68.24832	69.74595	1.715009	177.3328	79.86808	0.459091
DH_9_107	3.456232	77.13107	80.57557	4.324542	154.4194	74.47562	0.460729
DH_9_109	5.442989	68.92966	71.33423	2.189234	175.6802	80.43239	0.459315
DH_9_113	5.072312	69.61849	71.17177	1.636302	168.2429	77.87886	0.459158
DH_9_117	5.001858	78.03047	82.94173	3.906232	151.5615	73.96408	0.45927

Taxa	GY	AD	SD	ASI	PH	EH	EPO
DH_9_120	5.326396	69.06419	70.1453	1.350416	176.2577	81.42993	0.460234
DH_9_127	5.341992	71.49262	72.36475	1.145253	172.5038	79.25156	0.459299
DH_9_128	5.163557	68.95355	69.75544	1.136105	164.8411	73.97975	0.457069
DH_9_129	4.026164	73.25398	76.23907	2.636533	170.1762	75.60893	0.457051
DH_9_130	5.114303	69.74282	71.22604	1.470955	181.0459	85.13289	0.462278
DH_9_132	5.292453	68.81707	70.0118	1.322341	169.0983	80.4585	0.461364
DH_9_134	4.840631	70.09075	72.03914	1.907466	172.3361	76.66466	0.457087
DH_9_136	5.366676	69.93328	71.19133	1.402265	165.6753	75.51921	0.458159
DH_9_138	5.162886	70.03586	71.88686	1.795909	174.3071	79.56109	0.459287
DH_9_14	4.969165	70.37443	71.73566	1.650107	170.5617	78.96634	0.459895
DH_9_141	5.396616	70.51033	72.23221	1.878944	177.1469	79.31495	0.457154
DH_9_143	5.261388	70.61471	71.69434	1.295166	175.7198	79.55834	0.458673
DH_9_148	5.726778	69.73544	71.21194	1.62481	173.8707	80.97095	0.460252
DH_9_15	4.751376	68.90188	70.67184	1.776934	169.7056	79.0694	0.459492
DH_9_150	3.865557	73.65907	77.39399	3.127485	168.3469	73.65634	0.455202
DH_9_155	5.832455	67.78203	69.15387	1.530639	170.3509	80.19224	0.461188
DH_9_16	3.626178	72.37934	76.34008	3.279619	171.9274	73.38414	0.453917
DH_9_162	5.038369	70.43391	71.62485	1.377196	169.0864	75.29139	0.456617
DH_9_165	4.840786	71.27529	72.47601	1.399908	166.2173	76.30847	0.459245
DH_9_173	6.348152	69.16506	69.94123	1.202713	168.1157	75.17075	0.457199
DH_9_175	5.468766	69.64871	71.19295	1.637832	177.8586	82.34959	0.460696
DH_9_176	5.588716	69.08304	70.70036	1.665525	175.441	83.09027	0.461972
DH_9_18	5.874114	68.85229	69.58765	1.045787	178.4021	79.81816	0.458773
DH_9_183	5.689256	71.35493	71.99267	1.086761	179.4623	81.16355	0.458965
DH_9_186	5.396872	69.45041	70.33082	1.231026	171.1203	76.39256	0.457378
DH_9_188	3.896333	74.21414	77.27424	2.596728	163.7078	74.92212	0.458745
DH_9_19	5.51213	69.36059	71.14074	1.771189	181.1411	83.67148	0.460991
DH_9_190	5.723076	69.84589	70.93171	1.366245	175.6473	79.85561	0.45865
DH_9_191	5.194639	70.84872	72.48152	1.756115	181.7408	82.05702	0.458524
DH_9_2	6.223316	68.7946	70.0042	1.321979	170.5757	77.60911	0.458505
DH_9_21	5.021799	70.45554	71.62745	1.393128	183.6153	80.66081	0.457549
DH_9_23	5.422875	69.70685	71.3659	1.75847	174.9697	78.75309	0.458461
DH_9_25	5.365938	69.83535	71.4189	1.597006	165.1872	75.65809	0.45834
DH_9_28	5.201798	69.63916	70.91647	1.494124	171.9685	74.89411	0.455349

Taxa	GY	AD	SD	ASI	PH	EH	EPO
DH_9_32	5.626579	71.04685	72.55477	1.579727	174.2462	79.30028	0.459133
DH_9_34	5.311687	69.70472	71.14393	1.475498	169.5464	75.77954	0.456495
DH_9_37	4.972703	69.92829	71.85023	3.231823	165.5676	77.38756	0.460207
DH_9_38	4.984513	70.49705	71.32236	1.161798	171.7996	74.03744	0.454578
DH_9_41	4.348343	69.23994	71.43487	2.016423	172.5871	74.77393	0.454969
DH_9_42	5.023241	66.9896	69.03459	1.905455	171.8361	79.85355	0.460088
DH_9_44	4.568912	70.94789	73.47828	2.194717	167.4167	76.09176	0.458508
DH_9_45	5.002126	69.31836	70.33179	1.345763	172.428	84.93616	0.464835
DH_9_47	5.569925	70.53771	72.35579	1.78684	168.0751	74.38511	0.455518
DH_9_48	5.208244	69.98574	71.46089	1.397698	176.8228	81.96039	0.460573
DH_9_49	6.141304	68.87303	70.77274	1.868985	179.039	79.92294	0.458035
DH_9_50	5.521932	70.12214	71.74927	1.671525	176.4621	80.07051	0.458707
DH_9_52	5.351765	69.77903	70.62312	1.15589	177.0544	82.70015	0.46102
DH_9_55	5.568921	70.10894	72.39017	2.244451	177.0532	80.42055	0.458563
DH_9_56	4.822831	69.89627	71.34319	1.699039	180.8242	83.1757	0.460815
DH_9_57	5.268864	71.4626	72.563	1.462538	176.4094	81.41202	0.460383
DH_9_58	4.42605	70.41451	71.70399	1.505771	173.8538	79.39955	0.458863
DH_9_60	5.725361	69.54133	71.45971	1.823891	169.5905	79.36784	0.460556
DH_9_61	5.042923	68.69838	70.17098	1.481239	171.5448	78.5017	0.458826
DH_9_62	5.832991	68.17261	69.11387	1.114493	175.7767	81.49914	0.460709
DH_9_63	5.653861	69.48658	70.10011	1.205351	181.7449	84.03423	0.460738
DH_9_64	5.528685	70.75113	72.11459	1.54581	178.4511	82.93082	0.461141
DH_9_66	6.139277	69.12499	71.19752	1.992345	172.4513	76.91288	0.45768
DH_9_71	5.555931	69.05928	72.06416	2.625596	173.9455	80.32507	0.459595
DH_9_72	4.673659	68.60822	70.94408	2.215662	174.3254	79.02871	0.458818
DH_9_73	5.122271	70.36902	71.81417	1.531765	169.5184	76.85751	0.458163
DH_9_74	5.406217	70.08211	72.31122	1.980766	175.7341	82.5735	0.460907
DH_9_76	4.438983	71.78911	72.62976	3.37533	165.6206	77.80641	0.460418
DH_9_85	4.993487	69.06499	69.88784	1.286008	176.1624	80.2665	0.45927
DH_9_9	3.193622	77.68031	83.31386	5.445123	165.524	75.03398	0.457561
DH_9_93	5.579479	70.47834	71.07863	1.042544	172.597	77.85445	0.457419
DH_9_98	3.373023	77.54825	77.3862	2.316318	156.53	74.21727	0.457959
DH_9_99	5.192125	70.84844	72.15639	1.43498	176.719	79.85533	0.458802
