# Supplementary material for: Genome wide association mapping for heat tolerance in sub-tropical maize
Source: BMC Genomics. 2021 Mar 4;22:154. doi: 10.1186/s12864-021-07463-y (PMC7934507; doi:10.1186/s12864-021-07463-y)
Supplement: Supplementary file 9 — Additional file 9: Table S6. Best linear unbiased prediction value (BLUPs) for DH population under heat stress conditions across two years. [file 12864_2021_7463_MOESM9_ESM.pdf]

Table S6. Best linear unbiased prediction value (BLUPs) for DH population under heat stress conditions across two years.

Taxa	GY	ASI	EH	EPO
DH_1_10	3.628673	1.811302	59.6724	0.405195
DH_1_100	3.067595	3.443332	62.56908	0.429037
DH_1_101	3.453245	1.918202	60.32452	0.40105
DH_1_102	3.524636	1.942707	61.37385	0.408032
DH_1_103	3.450033	2.140855	61.47487	0.411845
DH_1_104	3.649859	2.382486	61.72382	0.413232
DH_1_106	3.520425	2.428393	60.06996	0.410916
DH_1_107	3.914999	2.455247	67.41328	0.435011
DH_1_109	4.125799	1.980735	65.15102	0.427468
DH_1_110	3.743752	2.309154	59.68457	0.404829
DH_1_111	3.880041	1.993092	62.42553	0.416579
DH_1_113	3.308113	2.231902	62.85071	0.421696
DH_1_114	3.749208	1.975193	63.3416	0.427679
DH_1_115	3.687467	2.082429	68.01817	0.437393
DH_1_116	3.384265	2.415329	59.88023	0.412431
DH_1_117	3.692044	1.915418	60.14564	0.404054
DH_1_118	3.937038	2.891461	62.53241	0.412208
DH_1_119	2.840299	2.785341	63.27928	0.428327
DH_1_12	4.090022	2.045234	63.99486	0.415797
DH_1_121	3.784669	2.490234	63.99651	0.424129
DH_1_123	3.326485	2.262272	62.2352	0.41204
DH_1_124	3.884793	2.114892	63.42736	0.419197
DH_1_126	3.64185	1.95607	66.23564	0.420567
DH_1_127	3.420722	3.754228	63.18503	0.421159
DH_1_129	3.730308	1.888012	65.5796	0.431937
DH_1_13	4.583131	1.91287	65.96237	0.425286
DH_1_130	3.737437	2.067068	64.2828	0.418781
DH_1_131	4.029098	2.00565	66.83636	0.439808
DH_1_132	3.7454	1.974384	62.47991	0.407776
DH_1_133	2.975601	2.778264	65.07794	0.426471

Taxa	GY	ASI	EH	EPO
DH_1_135	3.616281	2.136812	61.91415	0.413864
DH_1_136	3.670239	2.041993	63.34251	0.411247
DH_1_137	4.031613	2.165729	60.98618	0.402846
DH_1_138	3.200641	2.623493	65.31655	0.443915
DH_1_139	3.851284	2.953593	61.15254	0.409443
DH_1_140	3.383642	2.630377	60.96942	0.411519
DH_1_141	3.66707	2.070072	64.53147	0.429022
DH_1_143	4.393817	1.825632	66.84211	0.441344
DH_1_144	3.980009	2.020387	66.81894	0.435292
DH_1_145	3.23689	2.918324	63.84789	0.420188
DH_1_146	3.597855	1.939791	62.63757	0.414683
DH_1_147	3.227838	2.335134	64.63845	0.423094
DH_1_148	4.088042	2.03612	65.93994	0.430349
DH_1_149	4.077906	2.190929	68.00484	0.436168
DH_1_15	3.351	2.797445	62.18918	0.430494
DH_1_150	3.221675	2.526598	66.45064	0.430845
DH_1_151	3.539033	2.756142	61.01993	0.40722
DH_1_152	3.699606	1.717688	65.93445	0.430068
DH_1_153	3.53382	1.48274	63.27382	0.417764
DH_1_155	3.743981	1.86153	65.04102	0.424189
DH_1_156	3.791871	1.825034	68.20039	0.439953
DH_1_157	3.832267	2.222027	63.127	0.41366
DH_1_158	3.887456	2.437705	67.22851	0.443271
DH_1_159	3.685462	2.080397	63.38077	0.417353
DH_1_16	3.985849	1.949684	64.82886	0.421907
DH_1_160	4.440416	2.027825	63.20354	0.41235
DH_1_161	3.903446	2.191518	65.07466	0.421793
DH_1_164	3.985129	2.065827	66.12784	0.435318
DH_1_165	3.746018	2.097291	63.65941	0.433276
DH_1_166	3.913427	2.36115	62.75548	0.413671
DH_1_167	3.249037	2.645049	67.55472	0.436739
DH_1_168	3.663035	2.7951	64.19157	0.421917

Taxa	GY	ASI	EH	EPO
DH_1_169	3.422942	1.994046	60.51838	0.408021
DH_1_17	3.175273	2.734086	60.58566	0.408805
DH_1_170	3.951709	2.296466	60.47319	0.410975
DH_1_171	3.720226	2.540233	62.90026	0.427033
DH_1_172	3.381169	2.491076	65.3549	0.43041
DH_1_173	3.558795	2.566714	63.2251	0.422374
DH_1_174	3.746084	2.241213	59.89944	0.402123
DH_1_175	4.083309	2.271897	67.11899	0.430779
DH_1_176	3.496058	2.28146	60.38641	0.408103
DH_1_177	3.729683	1.966604	61.90688	0.414173
DH_1_178	4.172864	1.987716	62.33535	0.401336
DH_1_18	4.021143	2.094669	65.41382	0.426511
DH_1_180	3.179662	3.054224	67.90451	0.4387
DH_1_181	3.006068	2.202176	65.66534	0.428155
DH_1_182	3.116404	2.05786	62.90445	0.413238
DH_1_183	3.415607	2.029712	67.08725	0.435179
DH_1_184	3.48109	3.412141	58.55813	0.421759
DH_1_185	3.74947	2.285712	63.172	0.421201
DH_1_186	3.85367	1.929981	64.67773	0.41899
DH_1_187	3.270893	2.441233	62.82721	0.422596
DH_1_188	3.217685	2.490831	62.32109	0.417241
DH_1_189	3.849318	2.290981	63.04493	0.411486
DH_1_19	3.302947	2.143008	66.54476	0.437319
DH_1_190	3.895235	2.344076	62.00956	0.412646
DH_1_191	3.25672	1.895194	69.53335	0.458481
DH_1_192	3.533909	2.353122	61.37761	0.413252
DH_1_193	3.676688	1.935625	67.47703	0.43274
DH_1_194	3.563486	1.753409	63.87045	0.418731
DH_1_195	3.892846	1.960228	64.15318	0.415083
DH_1_196	3.938148	1.581014	62.79119	0.41085
DH_1_197	3.714618	2.221386	60.67947	0.411297
DH_1_199	3.723556	2.093682	63.24389	0.417788

Taxa	GY	ASI	EH	EPO
DH_1_2	3.310437	2.420369	65.18551	0.42609
DH_1_201	3.599593	1.920984	62.6204	0.424175
DH_1_202	3.540189	1.847741	61.71262	0.414472
DH_1_203	3.578058	2.342923	62.23366	0.409162
DH_1_204	3.751076	2.224269	66.03415	0.427133
DH_1_205	3.458397	2.012911	61.30971	0.410203
DH_1_206	3.545002	2.185128	63.52537	0.413675
DH_1_207	2.768038	2.483429	64.47357	0.440252
DH_1_208	3.886928	2.895836	69.05601	0.440656
DH_1_209	3.304327	2.25375	64.88903	0.427693
DH_1_210	3.541205	2.028665	64.35563	0.426502
DH_1_211	3.8254	1.817597	66.00526	0.43034
DH_1_212	3.721861	1.965207	63.73389	0.427148
DH_1_215	3.627972	2.424331	61.27448	0.408804
DH_1_216	3.487094	2.975543	63.78207	0.42598
DH_1_217	3.700681	2.070977	62.70479	0.413613
DH_1_218	3.564262	2.098934	64.4482	0.426947
DH_1_219	3.945684	2.335352	59.80455	0.394077
DH_1_22	3.543019	2.307461	66.29528	0.428657
DH_1_23	3.581373	2.453694	65.32907	0.419839
DH_1_24	3.974357	2.360719	67.373	0.434196
DH_1_25	3.526817	2.162196	60.84254	0.405121
DH_1_26	3.316832	2.320357	63.48861	0.429309
DH_1_27	3.365667	3.058494	67.65122	0.446845
DH_1_28	3.69755	2.057541	61.74669	0.417805
DH_1_29	3.338873	2.26759	62.67835	0.430592
DH_1_3	3.87473	2.24201	63.27616	0.413872
DH_1_32	3.672302	2.174055	60.52004	0.414782
DH_1_33	3.942921	2.18034	63.97181	0.424302
DH_1_35	4.190443	2.35661	63.36223	0.407257
DH_1_37	3.553291	2.400493	61.15604	0.407172
DH_1_38	3.100643	2.480854	64.50528	0.419899

Taxa	GY	ASI	EH	EPO
DH_1_39	3.119646	2.645189	62.71046	0.420841
DH_1_4	3.754431	2.022284	62.87976	0.40688
DH_1_41	3.307706	2.280298	62.59563	0.413178
DH_1_42	3.754438	1.92768	63.57074	0.422585
DH_1_44	3.6968	2.830295	65.01313	0.425522
DH_1_45	3.378477	1.985882	63.26663	0.415735
DH_1_46	3.474486	1.909674	66.31699	0.428059
DH_1_47	3.361275	2.561227	67.53813	0.435025
DH_1_49	3.99658	2.164518	61.39225	0.411347
DH_1_5	3.872171	2.479212	60.96364	0.414861
DH_1_50	3.677585	2.274229	64.48697	0.428451
DH_1_51	3.779686	2.431041	62.70178	0.412353
DH_1_52	3.682128	2.12101	62.56778	0.417947
DH_1_53	4.052678	2.151154	65.90195	0.434454
DH_1_55	3.63812	1.87709	65.29758	0.435785
DH_1_57	3.272329	2.755957	64.98412	0.428208
DH_1_58	3.642508	2.36901	65.1571	0.437953
DH_1_59	3.694963	2.876024	67.63485	0.445949
DH_1_6	3.890262	2.053007	63.53542	0.417573
DH_1_60	3.843867	1.903728	65.77188	0.433496
DH_1_61	4.047324	1.605671	64.88653	0.431024
DH_1_63	3.487264	2.405045	60.06176	0.415695
DH_1_65	3.796742	2.049556	64.40893	0.42209
DH_1_66	3.893364	2.388794	64.74063	0.422318
DH_1_67	3.770304	2.711748	63.99608	0.431662
DH_1_68	3.758851	1.990904	64.83622	0.421277
DH_1_69	3.484256	2.581812	65.20785	0.429788
DH_1_7	3.850705	2.343957	65.32836	0.426485
DH_1_70	4.050774	2.106419	65.73445	0.438024
DH_1_72	3.569195	2.000006	65.54594	0.42325
DH_1_73	3.378793	2.223681	65.7951	0.441191
DH_1_74	3.385	4.286301	62.20942	0.414898

Taxa	GY	ASI	EH	EPO
DH_1_77	3.432839	2.778574	61.23022	0.403152
DH_1_78	3.402902	1.892072	62.63026	0.429628
DH_1_79	3.336716	2.830491	60.36398	0.40204
DH_1_8	3.736752	2.408594	58.03287	0.396294
DH_1_80	3.861254	2.147311	63.22604	0.437085
DH_1_81	3.308093	1.980957	62.82874	0.419109
DH_1_82	3.766981	2.400394	63.51593	0.411849
DH_1_83	4.158843	2.219439	63.44721	0.423963
DH_1_84	3.777208	2.385262	63.36776	0.413833
DH_1_85	3.927704	1.990677	63.02012	0.421983
DH_1_86	3.919325	2.590079	66.4	0.429241
DH_1_87	3.268937	2.138716	63.6518	0.423416
DH_1_88	3.850651	1.920816	65.61132	0.440281
DH_1_9	3.473703	3.789539	61.50755	0.412576
DH_1_90	3.340912	1.955178	66.27039	0.437812
DH_1_91	3.290632	2.004887	65.79838	0.435632
DH_1_92	3.501904	2.272872	65.46688	0.422258
DH_1_93	3.507199	2.19891	61.43863	0.413234
DH_1_94	3.967106	2.147806	64.85457	0.429949
DH_1_95	3.687094	2.107663	63.45163	0.414915
DH_1_96	4.086579	1.971505	62.18114	0.4096
DH_1_97	3.689383	2.569761	63.66872	0.42703
DH_1_98	3.469662	2.443331	68.30976	0.435497
DH_1_99	3.219966	2.133497	69.02142	0.443418
DH_2_1	3.362976	3.084841	62.83302	0.408948
DH_2_102	3.957771	2.088572	63.61636	0.417286
DH_2_11	3.306506	2.856493	61.523	0.411307
DH_2_14	3.245177	3.357361	60.69161	0.412385
DH_2_15	3.783091	2.8796	62.29175	0.410346
DH_2_17	2.888202	3.173719	61.74994	0.409665
DH_2_18	3.378717	3.482729	65.10489	0.42901
DH_2_2	2.569245	5.367016	66.14679	0.421968

Taxa	GY	ASI	EH	EPO
DH_2_21	3.533318	3.211186	66.21647	0.433023
DH_2_24	3.059394	3.21321	63.6015	0.42367
DH_2_25	3.262888	3.026658	64.85349	0.419301
DH_2_27	3.775697	2.322942	67.91352	0.445602
DH_2_28	3.57129	2.747159	60.57979	0.405927
DH_2_3	2.809221	4.812468	61.84172	0.414673
DH_2_31	3.445542	2.969794	61.41648	0.413877
DH_2_33	3.262964	4.471246	63.29122	0.414103
DH_2_34	3.354718	3.528737	63.71036	0.413409
DH_2_35	3.031124	3.060303	63.03852	0.415795
DH_2_36	3.402859	2.92621	60.659	0.402444
DH_2_37	3.350083	3.013287	61.25074	0.400633
DH_2_38	3.338013	3.303344	62.36744	0.419838
DH_2_39	2.926283	3.777544	63.61684	0.413298
DH_2_42	3.406775	3.141395	63.73247	0.419614
DH_2_43	3.563034	3.659346	62.37603	0.410819
DH_2_48	3.08329	3.491113	64.20177	0.421543
DH_2_49	2.941754	3.111785	62.14123	0.415433
DH_2_5	3.301227	2.294849	60.6599	0.403478
DH_2_50	3.101751	4.081374	61.0193	0.406924
DH_2_53	2.767695	3.376262	66.59349	0.433871
DH_2_58	2.836656	3.653824	64.05011	0.421678
DH_2_59	2.75906	5.63453	65.26345	0.433857
DH_2_6	3.249606	3.204989	61.58269	0.411137
DH_2_61	3.563269	2.309892	62.44343	0.414626
DH_2_62	3.184387	3.509142	62.38608	0.421319
DH_2_65	2.547476	4.410324	66.84497	0.422807
DH_2_66	3.293626	3.537218	63.44526	0.414802
DH_2_67	2.777658	5.222932	63.73129	0.419249
DH_2_70	2.583334	4.489304	62.07739	0.408205
DH_2_71	2.853124	4.009394	60.40218	0.407897
DH_2_72	3.214235	2.152552	61.95327	0.411575

Taxa	GY	ASI	EH	EPO
DH_2_73	3.624164	3.024355	64.28666	0.419566
DH_2_74	3.205943	3.457872	61.94071	0.409734
DH_2_75	2.973077	3.574482	60.73185	0.41442
DH_2_78	2.588747	5.523833	61.75467	0.410924
DH_2_80	2.878416	5.24463	59.82357	0.399199
DH_2_81	3.002628	3.547087	62.83251	0.411511
DH_2_84	3.477335	3.67282	63.59638	0.420593
DH_2_85	3.33147	2.976987	64.46234	0.417494
DH_2_87	3.243555	2.951341	61.58106	0.405382
DH_2_88	3.296528	3.405692	63.70919	0.41139
DH_2_9	3.148608	2.722663	62.68859	0.413397
DH_2_90	3.652991	3.108212	64.94158	0.422165
DH_2_93	3.331376	2.99308	62.68717	0.413119
DH_2_96	3.429535	3.601728	61.8843	0.407346
DH_2_97	4.302346	2.499773	65.49594	0.419034
DH_2_98	3.422126	3.400628	62.18729	0.413933
DH_2_99	2.780664	2.967254	61.81433	0.412305
DH_3_1	3.940834	2.568998	63.17629	0.417453
DH_3_10	3.366662	4.202733	63.28983	0.414842
DH_3_101	2.845002	4.412906	60.51516	0.407449
DH_3_102	3.638678	2.925846	64.21374	0.425119
DH_3_103	3.94501	2.542816	63.38457	0.424355
DH_3_105	3.578173	2.158413	63.5737	0.417614
DH_3_106	3.864827	2.232866	62.75434	0.421322
DH_3_107	3.809438	2.142788	65.09514	0.417964
DH_3_108	3.823283	2.515892	63.46217	0.42037
DH_3_109	3.34831	2.734595	62.15765	0.405956
DH_3_11	3.951953	2.392716	62.98972	0.413402
DH_3_110	3.327811	3.744132	58.86236	0.400249
DH_3_111	3.256838	3.41054	64.02204	0.417773
DH_3_116	3.9495	2.341365	64.10528	0.415341
DH_3_123	3.43783	2.329622	63.34029	0.422979

Taxa	GY	ASI	EH	EPO
DH_3_124	3.627919	2.33396	63.85534	0.423305
DH_3_125	3.500483	2.343386	60.51913	0.408542
DH_3_126	4.56124	2.784782	63.05705	0.422147
DH_3_129	3.565342	2.846343	62.15359	0.419578
DH_3_13	3.650957	2.874768	65.09649	0.43091
DH_3_131	3.325662	3.004807	57.75212	0.394172
DH_3_135	3.030219	2.667695	63.0426	0.420555
DH_3_136	3.457219	2.489396	62.31511	0.420527
DH_3_137	3.847437	2.244738	62.81115	0.417863
DH_3_138	2.77817	3.501481	62.23748	0.417379
DH_3_139	3.44546	3.316772	61.40226	0.413121
DH_3_14	3.844865	2.440614	64.09607	0.416955
DH_3_142	3.579	2.126049	63.71259	0.437762
DH_3_143	3.163007	2.406379	61.60777	0.409772
DH_3_145	3.903361	2.951107	64.21707	0.416167
DH_3_147	3.361572	2.692335	62.87811	0.411113
DH_3_15	3.599274	2.531281	61.55604	0.41282
DH_3_151	4.043237	3.626304	62.74813	0.413483
DH_3_16	2.659409	3.133585	59.76143	0.412354
DH_3_18	3.775188	2.90037	64.56851	0.433762
DH_3_19	3.450413	3.050611	61.6241	0.411832
DH_3_2	4.226735	2.452334	65.12008	0.420701
DH_3_20	3.231122	3.049733	60.98303	0.411118
DH_3_22	3.229446	2.855086	62.70668	0.415153
DH_3_23	3.142002	2.405649	62.25455	0.418971
DH_3_26	3.528145	2.946945	61.9478	0.409187
DH_3_27	3.523257	3.065437	60.71287	0.403184
DH_3_29	3.552334	2.513441	61.49496	0.409228
DH_3_3	3.349615	2.673335	62.83845	0.420024
DH_3_30	4.261299	3.007382	61.33814	0.412976
DH_3_31	2.968765	3.823639	61.02152	0.408546
DH_3_32	3.250345	4.223073	60.42584	0.414146

Taxa	GY	ASI	EH	EPO
DH_3_34	3.127112	2.812474	62.34078	0.419532
DH_3_36	3.144294	2.970433	64.01651	0.413715
DH_3_38	3.488847	2.211763	63.54387	0.413613
DH_3_39	3.615185	3.157239	60.48826	0.403854
DH_3_4	3.634756	2.105949	64.0052	0.416598
DH_3_41	3.760821	2.608799	64.78006	0.428799
DH_3_43	3.8978	2.29997	61.80005	0.407257
DH_3_46	3.712201	2.186529	63.6623	0.421564
DH_3_47	3.451318	2.417993	59.16485	0.401597
DH_3_48	3.126785	2.363464	61.38845	0.413984
DH_3_5	3.064027	3.471434	61.58035	0.411577
DH_3_50	3.755516	3.490098	61.36344	0.406426
DH_3_53	3.534007	2.081932	59.29435	0.3942
DH_3_56	3.957358	3.096757	58.99559	0.402312
DH_3_57	3.915534	2.397612	62.4677	0.414311
DH_3_58	3.858342	2.351461	62.47485	0.411338
DH_3_59	3.528556	2.879671	62.1402	0.41349
DH_3_6	3.468717	2.787373	62.38429	0.412802
DH_3_60	3.331817	2.65372	62.9204	0.414261
DH_3_61	3.844154	2.481434	60.77921	0.404999
DH_3_62	3.625708	2.513174	63.41311	0.426863
DH_3_63	3.729271	2.685261	61.49857	0.411723
DH_3_64	3.585865	2.378739	62.16303	0.409754
DH_3_65	3.719763	4.214908	65.00249	0.42455
DH_3_66	3.767694	2.81191	61.2126	0.401566
DH_3_67	4.091311	2.291118	64.15562	0.429346
DH_3_68	3.742026	3.527491	64.16867	0.421823
DH_3_69	3.604351	2.060495	63.89196	0.423708
DH_3_7	3.135668	2.923329	59.67544	0.418248
DH_3_72	3.776576	2.856464	64.93851	0.425239
DH_3_74	4.228002	2.294408	61.90645	0.401766
DH_3_75	3.562842	3.476344	64.44942	0.424853

Taxa	GY	ASI	EH	EPO
DH_3_77	3.432193	3.85153	60.13698	0.409567
DH_3_80	3.925768	2.307219	62.94423	0.41411
DH_3_81	3.164526	3.433507	59.6998	0.395379
DH_3_82	3.032413	2.764602	61.55565	0.408074
DH_3_83	3.264067	2.858586	63.74357	0.423601
DH_3_84	2.958474	3.264519	57.99593	0.401665
DH_3_85	3.381824	2.087741	62.86207	0.417225
DH_3_86	3.548413	2.315554	61.32113	0.420907
DH_3_87	3.861919	2.392808	59.26224	0.405767
DH_3_88	3.333123	2.451275	62.45569	0.419719
DH_3_89	3.68041	0.249656	59.00299	0.397337
DH_3_9	3.317425	2.824449	63.04275	0.420068
DH_3_91	3.513237	2.001685	65.24212	0.42364
DH_3_94	3.884052	1.896739	65.65414	0.419428
DH_3_95	3.19446	3.332176	60.10386	0.405905
DH_3_96	3.770533	1.658887	66.96702	0.431222
DH_3_98	2.819004	2.653851	61.11721	0.407141
DH_3_99	3.585487	2.148468	63.57457	0.408073
DH_4_11	3.617874	2.664051	63.65717	0.416646
DH_4_12	3.546884	2.616007	66.24715	0.427647
DH_4_13	3.686254	2.282953	62.91121	0.419604
DH_4_14	3.613632	2.913308	62.34753	0.421461
DH_4_19	3.351382	2.955332	62.33859	0.406764
DH_4_20	3.28192	2.340286	61.64375	0.40274
DH_4_22	3.785714	2.435876	64.49501	0.415669
DH_4_23	4.558431	2.366485	64.95668	0.419769
DH_4_24	3.782811	2.303366	63.65031	0.416014
DH_4_25	4.145746	2.285857	64.16913	0.418076
DH_4_26	3.584894	2.173468	63.00983	0.409843
DH_4_27	3.532276	1.967528	66.6892	0.42677
DH_4_28	3.48565	2.42227	63.79852	0.43644
DH_4_29	3.626615	2.493494	62.97471	0.41232

Taxa	GY	ASI	EH	EPO
DH_4_3	3.256534	2.874147	63.35614	0.418786
DH_4_30	3.400347	2.491442	62.27845	0.413035
DH_4_31	3.15058	2.950155	60.78233	0.40645
DH_4_32	3.611524	2.293146	65.11001	0.428345
DH_4_33	3.796003	2.207781	63.87734	0.419656
DH_4_34	3.708816	2.612874	66.27942	0.422123
DH_4_35	3.138255	2.271215	65.84298	0.420793
DH_4_36	3.778003	2.797866	62.81067	0.42071
DH_4_37	3.451008	2.472959	64.01481	0.41767
DH_4_38	3.970754	2.138036	62.09205	0.412699
DH_4_39	3.389215	2.999446	61.4593	0.405225
DH_4_4	3.457354	3.428941	61.10582	0.406819
DH_4_42	3.031877	2.09805	67.1174	0.428058
DH_4_44	3.490398	2.26376	61.07595	0.401854
DH_4_45	3.806302	2.584648	61.2803	0.399826
DH_4_46	3.483427	2.576971	60.50883	0.387474
DH_4_47	4.31577	2.238477	65.34565	0.428169
DH_4_49	4.314524	1.943209	66.01935	0.428703
DH_4_53	3.486683	2.793454	64.26019	0.42107
DH_4_54	4.094005	3.31389	63.27651	0.420454
DH_4_8	3.722301	2.499232	63.3331	0.417645
DH_4_9	2.883037	4.615937	62.50718	0.417357
DH_5_1	2.994965	3.403115	64.31188	0.427326
DH_5_10	3.239274	2.261201	66.69345	0.434689
DH_5_11	2.936068	4.604891	63.53857	0.416886
DH_5_12	4.025586	2.058308	64.2532	0.414901
DH_5_13	3.630156	1.613908	63.0961	0.409725
DH_5_15	3.468338	2.728129	61.90371	0.41937
DH_5_17	3.052715	5.256639	61.46301	0.413057
DH_5_2	3.575775	2.264375	66.00412	0.42571
DH_5_21	2.674647	3.529692	63.52649	0.415043
DH_5_22	3.327811	2.31367	64.61076	0.417035

Taxa	GY	ASI	EH	EPO
DH_5_23	3.62982	3.615346	63.28755	0.406927
DH_5_26	3.212428	2.393305	63.96958	0.420264
DH_5_27	3.121921	3.594308	65.43268	0.427002
DH_5_28	2.839073	4.950668	64.52551	0.424119
DH_5_3	3.061745	2.427188	64.19411	0.419373
DH_5_33	3.354711	2.438284	62.17598	0.406697
DH_5_35	3.696966	3.971061	68.60891	0.438155
DH_5_36	3.484591	4.031103	64.89276	0.428285
DH_5_37	3.279996	3.027712	61.72049	0.427487
DH_5_38	3.428949	2.443454	59.48999	0.40095
DH_5_4	3.933	2.26925	68.15006	0.433931
DH_5_40	3.343967	2.980643	65.11013	0.423742
DH_5_41	3.242191	2.862341	64.03407	0.41334
DH_5_43	2.932706	4.955744	61.288	0.415949
DH_5_44	3.27393	2.943288	62.85986	0.414832
DH_5_45	2.79505	2.531559	65.2822	0.415482
DH_5_47	4.116266	2.451895	65.20397	0.426973
DH_5_48	3.421156	2.868567	64.35112	0.422622
DH_5_5	3.06903	3.489827	58.60002	0.409426
DH_5_51	3.425539	1.781791	65.8132	0.426951
DH_5_53	3.366342	2.502981	65.10158	0.423858
DH_5_56	3.524471	2.764386	63.47186	0.41599
DH_5_57	4.066557	2.564825	65.13552	0.418719
DH_5_58	3.446995	2.651018	64.1408	0.424822
DH_5_59	2.908107	2.76366	65.03857	0.424305
DH_5_60	2.872689	2.612332	62.91432	0.422049
DH_5_62	3.098091	2.187004	62.81821	0.411872
DH_5_63	3.671421	2.489906	64.50994	0.420007
DH_5_65	2.709302	2.914031	61.46905	0.403949
DH_5_67	3.976316	2.265781	66.48442	0.428351
DH_5_69	2.754494	2.641848	59.67285	0.396909
DH_5_70	3.678288	2.009172	69.42814	0.439525

Taxa	GY	ASI	EH	EPO
DH_5_73	3.387039	2.911175	63.03119	0.417117
DH_5_74	3.569406	2.621283	63.72105	0.413387
DH_5_75	2.57617	3.552968	65.37406	0.424951
DH_5_76	3.423234	2.55498	65.99804	0.434312
DH_5_77	3.272438	2.476042	66.27102	0.428301
DH_5_8	3.723481	2.417944	62.09821	0.414034
DH_5_9	3.028722	4.289767	63.44394	0.418828
DH_6_1	3.23103	2.582602	64.87364	0.428354
DH_6_100	3.543799	2.111671	60.05407	0.400265
DH_6_101	3.257573	3.128854	62.41112	0.423449
DH_6_105	3.981247	2.770235	62.92935	0.414054
DH_6_106	3.634119	2.844497	60.08139	0.400467
DH_6_108	3.537446	2.756382	60.21818	0.40157
DH_6_112	3.443402	3.148333	62.39937	0.425792
DH_6_113	3.273939	3.427539	60.79073	0.410653
DH_6_114	2.996773	3.877153	59.13973	0.406287
DH_6_116	3.591407	2.446091	63.9448	0.427126
DH_6_120	3.31215	2.37077	59.61627	0.401073
DH_6_13	4.093278	2.121017	62.41076	0.413325
DH_6_14	3.723328	2.380389	61.21691	0.404599
DH_6_15	3.055283	2.183792	62.6483	0.424571
DH_6_16	3.578405	2.823607	59.2597	0.392442
DH_6_17	3.527825	2.355005	63.01641	0.419098
DH_6_19	3.567252	3.888988	62.49417	0.406428
DH_6_20	3.147336	3.396861	60.82989	0.403402
DH_6_22	3.812859	2.710011	61.4414	0.402262
DH_6_23	3.256369	2.517816	64.21457	0.417442
DH_6_26	3.305813	2.659153	60.33328	0.404898
DH_6_28	3.295141	2.796746	64.30709	0.426032
DH_6_29	3.061746	1.826076	61.30164	0.406316
DH_6_30	3.490808	3.266534	58.96293	0.399857
DH_6_31	3.05028	2.409809	59.42685	0.403722

Taxa	GY	ASI	EH	EPO
DH_6_33	3.227874	3.423805	58.82191	0.397523
DH_6_35	3.118722	3.457357	60.33883	0.408947
DH_6_38	3.208211	3.087623	59.19627	0.39752
DH_6_4	3.641802	2.998377	62.13498	0.411501
DH_6_40	3.253412	2.62676	61.87447	0.420091
DH_6_41	3.253031	2.998976	62.73203	0.41124
DH_6_43	3.499022	3.478301	59.90766	0.40459
DH_6_45	3.349616	2.895583	63.61464	0.416808
DH_6_46	3.407368	2.611669	64.39621	0.426895
DH_6_5	3.19777	3.317518	59.74849	0.407511
DH_6_52	3.531899	2.70397	58.50646	0.398354
DH_6_57	3.839686	2.601045	60.5119	0.398714
DH_6_6	3.286062	3.210706	59.30625	0.400249
DH_6_60	3.25252	2.724586	61.22853	0.403801
DH_6_61	3.560026	2.605907	58.9012	0.399534
DH_6_64	3.1422	3.06816	60.53243	0.41013
DH_6_65	3.339095	2.338635	60.95801	0.401325
DH_6_68	3.083375	2.680379	61.20364	0.407688
DH_6_7	3.718651	2.622173	65.0576	0.416871
DH_6_71	3.785177	2.795994	61.63983	0.41189
DH_6_74	2.770698	3.492922	58.62373	0.403203
DH_6_75	3.739102	2.758371	62.68866	0.420359
DH_6_76	3.353503	2.498558	62.04405	0.409146
DH_6_79	3.190318	2.790654	61.75898	0.41005
DH_6_81	3.497032	2.301634	59.75816	0.397225
DH_6_83	3.185871	3.081866	62.67783	0.414794
DH_6_84	2.859848	2.906095	59.89333	0.402508
DH_6_85	3.501607	3.026382	64.94566	0.419562
DH_6_86	3.216195	3.254408	61.41011	0.408363
DH_6_90	3.66949	2.561335	61.39919	0.413303
DH_6_91	3.597212	2.329034	61.12071	0.407387
DH_6_93	3.276113	2.907725	62.52604	0.411446

Taxa	GY	ASI	EH	EPO
DH_6_94	3.333436	2.438422	64.18244	0.420497
DH_6_95	3.261162	2.552953	59.19369	0.391458
DH_6_97	3.011981	3.284192	63.1224	0.411022
DH_6_98	3.49973	2.451086	68.14736	0.467905
DH_7_10	3.454834	2.312163	63.65594	0.427732
DH_7_102	3.290401	2.493998	65.21714	0.434824
DH_7_12	3.567148	2.66672	64.56458	0.430121
DH_7_14	3.206053	2.735433	62.11582	0.417081
DH_7_16	3.707694	2.297127	62.66054	0.418155
DH_7_2	3.478955	3.199327	59.92283	0.405681
DH_7_21	3.642664	2.367763	65.73489	0.424819
DH_7_22	3.66601	2.35178	66.18707	0.427539
DH_7_24	3.629893	2.544045	61.02244	0.402878
DH_7_25	3.212912	2.401583	62.52422	0.421717
DH_7_27	3.56841	2.147393	61.99258	0.410622
DH_7_28	2.921226	3.026039	59.37855	0.411274
DH_7_29	3.565102	2.794135	61.54358	0.415025
DH_7_32	3.246834	3.081154	59.88071	0.399604
DH_7_33	3.037868	2.317441	59.8566	0.40423
DH_7_34	2.755129	2.949424	59.64324	0.411213
DH_7_37	2.794312	2.817629	62.53792	0.430485
DH_7_4	3.562997	2.463827	61.5785	0.405303
DH_7_43	3.552638	4.170245	65.12114	0.425333
DH_7_44	3.177925	2.805324	62.13812	0.420088
DH_7_46	3.591528	2.495321	62.78399	0.416795
DH_7_49	3.335116	2.537401	59.62699	0.409994
DH_7_50	2.828335	2.555791	64.94798	0.438462
DH_7_51	3.344893	2.824568	60.85166	0.405624
DH_7_52	3.188017	2.538983	61.91487	0.420541
DH_7_53	3.365986	2.771509	63.88708	0.4223
DH_7_54	3.908463	2.296491	67.08665	0.425929
DH_7_57	3.502137	2.548694	64.00653	0.423991

Taxa	GY	ASI	EH	EPO
DH_7_58	3.808064	2.354458	64.81427	0.423035
DH_7_60	3.786589	2.319883	65.35863	0.428644
DH_7_62	3.086103	3.747412	60.79741	0.423331
DH_7_64	3.930045	2.409027	66.66853	0.431908
DH_7_65	3.269936	3.260718	58.71983	0.411255
DH_7_66	3.879719	1.961854	64.76636	0.427665
DH_7_68	3.080418	2.267272	60.85039	0.406302
DH_7_69	3.301819	2.866914	60.37023	0.413722
DH_7_7	3.128521	2.587058	65.46774	0.428721
DH_7_70	2.834295	3.430248	58.26645	0.404622
DH_7_72	3.564637	2.503747	63.14499	0.413668
DH_7_73	3.719603	2.28477	60.65788	0.406814
DH_7_79	3.080243	3.243243	62.5729	0.408602
DH_7_8	3.221643	2.835458	61.69518	0.424916
DH_7_85	3.269	2.36822	61.93576	0.407279
DH_7_86	2.932564	2.464153	63.88101	0.435391
DH_7_87	3.348602	2.158384	61.73406	0.426012
DH_7_89	3.544669	2.524	62.34117	0.426078
DH_7_90	3.755271	1.925324	67.20611	0.430153
DH_7_91	3.055004	2.499813	62.14959	0.421637
DH_7_96	3.883712	2.681573	62.94788	0.41686
DH_7_97	4.086341	2.115507	62.20287	0.415871
DH_7_98	3.671691	2.446039	64.93265	0.424856
DH_9_1	3.480183	3.556386	60.77458	0.400053
DH_9_10	3.210755	2.516429	60.47168	0.405857
DH_9_101	3.519105	2.31895	62.46272	0.407337
DH_9_105	3.287483	3.083575	61.8662	0.406975
DH_9_107	3.273564	5.452183	62.92554	0.417274
DH_9_109	2.987295	3.782227	60.31138	0.406836
DH_9_113	3.823917	3.350359	59.50238	0.397378
DH_9_117	3.258667	5.239339	61.07414	0.411228
DH_9_120	2.868883	3.18369	58.03913	0.403904

Taxa	GY	ASI	EH	EPO
DH_9_127	3.219731	2.157058	60.15113	0.419461
DH_9_128	3.053491	3.202397	57.41019	0.395215
DH_9_129	3.334003	3.31897	61.89188	0.409874
DH_9_130	3.310111	3.008308	58.99366	0.401455
DH_9_132	2.977094	2.744742	55.41039	0.385001
DH_9_134	3.572758	2.187659	61.75518	0.423724
DH_9_136	3.006818	3.351677	58.17781	0.391774
DH_9_138	3.333098	2.969784	60.76804	0.405247
DH_9_14	3.326278	2.164955	59.50806	0.396896
DH_9_141	3.4974	2.482167	66.65364	0.431181
DH_9_143	3.26433	2.274163	62.83754	0.416364
DH_9_148	3.214602	2.356164	60.19621	0.406819
DH_9_15	3.873638	2.597711	61.24561	0.409321
DH_9_150	3.52874	2.016083	65.20675	0.424702
DH_9_155	3.699242	2.867035	59.78093	0.398521
DH_9_16	3.201026	2.81546	62.5935	0.412521
DH_9_162	3.318007	2.174755	64.00715	0.431848
DH_9_165	2.979361	2.88589	57.23877	0.387617
DH_9_173	3.848116	3.004173	62.08897	0.410623
DH_9_175	3.338957	2.516814	61.16086	0.412443
DH_9_176	3.335968	2.644859	61.59798	0.413807
DH_9_18	3.519344	2.676318	62.51443	0.409481
DH_9_183	3.146587	2.929748	58.89229	0.403092
DH_9_186	3.230698	3.357731	56.56	0.380672
DH_9_188	3.330193	2.646075	60.47921	0.403703
DH_9_19	3.949175	2.499018	61.21718	0.40801
DH_9_190	3.747133	1.42424	60.95263	0.404805
DH_9_191	3.379206	2.874049	60.89246	0.403874
DH_9_2	3.621304	2.285504	62.6996	0.414538
DH_9_21	3.122114	2.896168	58.38115	0.401701
DH_9_23	3.681734	2.559883	63.55359	0.413433
DH_9_25	3.631766	2.11374	60.35798	0.401558

Taxa	GY	ASI	EH	EPO
DH_9_28	3.361849	3.428906	57.65681	0.390193
DH_9_32	3.900714	2.378551	64.92844	0.424447
DH_9_34	3.325842	3.079799	61.32801	0.408269
DH_9_37	3.252214	2.356889	61.2361	0.41731
DH_9_38	3.954799	2.996841	62.92371	0.424618
DH_9_41	3.482048	1.981748	61.92418	0.411902
DH_9_42	3.202769	2.387245	62.93094	0.411786
DH_9_44	3.210389	3.217771	60.10078	0.407133
DH_9_45	3.824998	2.59411	62.56713	0.414692
DH_9_47	3.706977	2.186477	60.92694	0.403759
DH_9_48	3.67504	2.14445	60.50919	0.403961
DH_9_49	3.708415	2.672289	60.61379	0.400982
DH_9_50	3.81068	2.370344	64.40853	0.425815
DH_9_52	3.51419	2.250782	61.06827	0.412688
DH_9_55	3.238174	2.898127	59.68157	0.405153
DH_9_56	3.464865	2.670369	61.32967	0.415143
DH_9_57	3.325517	2.633711	61.3012	0.407253
DH_9_58	3.218325	3.397989	57.99486	0.398401
DH_9_60	3.662623	3.045743	59.97536	0.405204
DH_9_61	3.614245	2.597528	60.92226	0.400651
DH_9_62	3.288688	2.990368	62.86407	0.412499
DH_9_63	3.240083	2.41442	61.01377	0.404963
DH_9_64	2.944843	2.486328	60.97763	0.403786
DH_9_66	3.857358	2.820383	60.09433	0.402723
DH_9_71	3.215455	2.525105	56.87587	0.388114
DH_9_72	2.751012	5.069524	60.40307	0.403166
DH_9_73	2.908018	3.110277	57.95215	0.403289
DH_9_74	3.523811	2.50182	64.81076	0.433242
DH_9_76	3.119539	2.553516	58.94744	0.404107
DH_9_85	3.546701	2.070003	61.64964	0.403857
DH_9_9	3.175177	3.175519	61.78463	0.421178
DH_9_93	4.103484	2.073259	63.71554	0.415175

Taxa	GY	ASI	EH	EPO
DH_9_98	3.150436	2.960067	60.59637	0.398123
DH_9_99	3.455617	2.332247	65.34697	0.429444
DH_10_1	3.050076	2.437182	63.5479	0.426882
DH_10_10	3.63518	2.76405	62.21017	0.419624
DH_10_103	3.63534	2.401198	66.36888	0.427613
DH_10_104	3.310697	2.52938	62.26409	0.419118
DH_10_12	3.192118	2.703239	59.94744	0.404629
DH_10_13	3.926283	3.634181	64.38781	0.426643
DH_10_19	3.182556	3.12921	59.01922	0.407237
DH_10_2	3.390388	1.965738	61.86462	0.413717
DH_10_26	3.485585	2.839788	64.88034	0.42972
DH_10_41	3.393786	2.13396	64.38166	0.428702
DH_10_42	3.850331	2.303647	60.45799	0.403255
DH_10_43	3.582684	2.796071	59.33634	0.404455
DH_10_44	3.619468	2.328032	60.95879	0.41249
DH_10_46	3.302443	2.78246	60.59707	0.404029
DH_10_49	3.233466	3.349937	62.72707	0.413312
DH_10_50	3.602712	2.669959	60.53608	0.41249
DH_10_52	3.249232	2.682474	62.12487	0.412086
DH_10_53	2.862609	2.507291	56.62088	0.393271
DH_10_54	3.194855	3.084058	61.58697	0.414167
DH_10_62	3.612599	2.549906	60.23917	0.400205
DH_10_64	3.111443	2.468074	61.0787	0.414004
DH_10_65	2.881496	3.121976	62.44983	0.422133
DH_10_67	3.510521	2.044796	62.53267	0.418913
DH_10_68	3.655682	2.654059	63.44828	0.421031
DH_10_7	3.336001	2.507826	61.99478	0.415947
DH_10_74	3.506978	2.563904	65.24135	0.429213
DH_10_78	3.648285	4.211413	59.91631	0.401625
DH_10_84	3.026122	2.252588	61.61801	0.418895
DH_10_86	3.392523	2.067353	61.78272	0.415173
DH_10_87	3.318702	2.11078	61.4462	0.417153
DH_10_9	3.326905	2.697614	62.46787	0.415422
DH_10_91	3.418104	4.302704	61.9316	0.413703

Taxa	GY	ASI	EH	EPO
DH_10_93	3.491171	2.076	64.26032	0.422398
DH_10_97	3.04829	2.868162	61.05055	0.419774
DH_10_98	3.580551	3.057338	61.54354	0.415608
